# Two extremely rare new species of fossorial salamanders of the genus *Oedipina* (Plethodontidae) from northwestern Ecuador

**DOI:** 10.7717/peerj.9934

**Published:** 2020-10-02

**Authors:** Carolina Reyes-Puig, David B. Wake, Ramachandran Kotharambath, Jeffrey W. Streicher, Claudia Koch, Diego F. Cisneros-Heredia, Mario H. Yánez-Muñoz, Santiago Ron

**Affiliations:** 1 Colegio de Ciencias Biológicas y Ambientales COCIBA, Universidad San Francisco de Quito, Campus Cumbayá, Quito, Ecuador; 2 Museo de Zoología & Laboratorio de Zoología Terrestre, Instituto de Diversidad Biológica Tropical iBIOTROP, Universidad San Francisco de Quito, Campus Cumbayá, Quito, Ecuador; 3 Unidad de Investigación, Instituto Nacional de Biodiversidad (INABIO), Quito, Ecuador; 4 Fundación EcoMinga, Baños, Ecuador; 5 Museum of Vertebrate Zoology and Department of Integrative Biology, University of California, Berkeley, California, United States of America; 6 Department of Zoology, Central University of Kerala, Kerala, India; 7 Department of Life Sciences, The Natural History Museum, London, United Kingdom; 8 Leibniz-Institut für Biodiversität der Tiere, Zoologisches Forschungsmuseum Alexander Koenig, Bonn, Germany; 9 Department of Geography, King’s College London, London, United Kingdom; 10 Museo de Zoología, Pontificia Universidad Católica del Ecuador, Quito, Ecuador

**Keywords:** Cryptic diversity, *Oedopinola*, Endemism, Foothills of western Ecuador, Choco

## Abstract

We describe two new species of salamanders of the genus *Oedipina*, subgenus *Oedopinola*, from two localities on the northwestern foothills of Ecuador, at elevations between 921 and 1,067 m. These are the southernmost members of the genus. We examined different museum collections and we found just three specimens of *Oedipina* from Ecuador, obtained throughout the history of herpetological collections in the country. We identify two of the three specimens as new species, but refrain from assigning a specific identity to the third, pending further study. *Oedipina villamizariorum* sp. n. is a medium-sized member of the genus, with a narrow, relatively pointed head and blunt snout; dorsolaterally oriented eyes, moderate in size; and digits that are moderately long and having pointed tips. *Oedipina ecuatoriana* sp. n., somewhat larger, has a narrow head and broadly rounded snout; this new species differs from all known *Oedipina* by the distinctive presence of paired prefrontal bones and a reduced phalangeal formula: 0-0-1-0; 0-1-2-1-1. We provide detailed descriptions of the osteology of both new species. Finally, we present a phylogenetic hypothesis for the genus, including one of the two new species, based on partial sequences of mitochondrial DNA.

## Introduction

Plethodontid salamanders from the American tropics are among the most diverse amphibians, with their radiation concentrated in Middle America (Rovito et al., 2015; Wake, 1987). With the exception of a few members of the family Salamandridae in southeast Asia, only plethodontids occupy tropical environments. Although they are a deeply nested clade, tropical plethodontids have experienced an extensive adaptive radiation, largely centered in Mexico and Central America, and now constitute more than 40% of all species of salamanders. Given the vast discrepancy in size between Middle and South America, it is at first surprising to learn that only 13% of the tropical salamanders live in South America (Frost, 2020). This is partly an artifact, because salamanders in South America have been understudied and recent research suggests that many more species remain to be described (Jaramillo et al., 2020), but salamanders continue to be discovered in Mexico and Central America as well (Parra Olea et al., 2020). Only two of 22 major clades of the Tribe Bolitoglossini (the tropical plethodontids) reach South America and neither of them is endemic (Rovito et al., 2015). Nevertheless, at the level of species much remains to be learned, and perhaps there are still surprises in store.

The lower number of salamander species in South versus Middle America generally is attributed to their relatively late arrival, after the main diversification of tropical salamanders had occurred (Rovito et al., 2015). This is reflected in the decreasing latitudinal diversity. The small country of Guatemala, less than 40% the size of Ecuador, has 65 species of plethodontids, belonging to eight genera (Frost, 2020). The majority are *Bolitoglossa*, but six of its seven subgenera are represented, as well as two of the three subgenera of *Oedipina* (Keferstein, 1868). There are 30 plethodontids in Panama, but only two genera, *Bolitoglossa* and *Oedipina*, with two subgenera of each represented. In contrast, the first general survey of South American salamanders (Brame & Wake, 1963) reported 17 species, 15 *Bolitoglossa* (Duméril, Bibron & Duméril, 1854), and two *Oedipina*. Since that time more than 30 South American *Bolitoglossa* have been described, but the number of *Oedipina* has remained constant, and both genera are represented by only a single subgenus, which also occurs in Panama. Thus, despite the 40 species (doubtless with many more to come) on the continent, the phylogenetic disparity between Middle and South America is dramatic.

Virtually all attention since 1963 has been given to *Bolitoglossa* in South America and *Oedipina* has nearly been ignored. We anticipate that this paper will initiate studies of South American *Oedipina*. While *Oedipina* has long been known from South America, very few specimens from very few localities have been reported (Acosta-Galvis et al., 2020; Brame, 1968). One of the first members of the genus to be discovered, *Oedipina parvipes*, is from the Rio Magdalena River valley in northwestern Colombia (Peters, 1879). It subsequently was reported from Panama and Ecuador (Dunn, 1926), an unlikely large range for any tropical salamander but especially a secretive fossorial form. Brame & Wake (1963) re-identified the single specimen from Ecuador (Paramba, northwestern Ecuador) as *Oedipina complex*, type locality central Panama, an even less likely distribution for a smaller species than *O. parvipes*. Also included in *O. complex* was a single specimen from Isla Gorgona, in the Pacific Ocean off the central coast of Colombia.

Specimens of *Oedipina* are relatively rarely encountered. They are characterized by a slender, elongated body (Brame, 1968; McCranie, Vieites & Wake, 2008) coupled with short limbs and a tail that can be twice body length, and secretive habits. *Oedipina* is distributed from southern Mexico to northwestern South America, between 0 and 2,320 m (Brame, 1968; highest elevation MVZ 190849, *Oedipina altura*). At the time of Brame’s review (which synonymized a number of species) 15 species were recognized; currently there are 38 species unevenly distributed among three subgenera, *Oedipina* (22 species), *Oeditriton* (3 species) and *Oedopinola* (13 species). *Oedipina* and *Oeditriton* are in general smaller, slenderer and have longer to much longer tails and shorter limbs than *Oedopinola* and cannot be distinguished from each other morphologically. They have the highest numbers of trunk vertebrae (as high as 23 in *O. taylori*, Brame, 1968) recorded in any tropical plethodontid, and none have fewer than 19 (most have 20). *Oedopinola* includes the northernmost (*O. elongata*, the only Mexican member of the genus) and southernmost members of *Oedipina*, and the only ones in South America. The taxon includes the shortest-bodied (most have 18 trunk vertebrae and none have more than 19), longest-legged and most robust members of *Oedipina*, and most have well-ossified skulls that are unusually solidified (García-París & Wake, 2000), possibly associated with their fossorial and burrowing habits. Some *Oedopinola* (e.g., *O. carablanca*) have broad, almost *Bolitoglossa*-like manus and pes, but others have very thin, narrow structures that show reductions in phalangeal number and complexity.

Most species of the genus are known from relatively few individuals (Brame, 1968; Brodie, Acevedo & Campbell, 2012; McCranie, Vieites & Wake, 2008), but some of the Costa Rican species were once very common in lower cloud forest situations. The fossorial nature of most members of this taxon may explain the limited knowledge of distribution, but the vast majority of species occur in Middle America, south and east of the Isthmus of Tehuantepec (e.g.,11 in Honduras, 12 in Costa Rica) (AmphibiaWeb, 2020).

The elusive nature of species of *Oedipina* accounts for our lack of knowledge of their life history, ecology, and behavior. Most are thought to be fossorial. They are encountered in forested areas under and in logs and rotted tree stumps, often in moss matts covering tree stumps and road banks. They can be especially common locally in the rich volcanic soils of the cloud forests of Central America. They also are found under the bark of logs and tree trunks (especially true of species of *Oedopinola*). A few species, such as *O.* (*Oedopinola*) *alleni*, can be found at night climbing on vegetation, and several have been found foraging on the forest floor at night (Köhler, 2011). Sometimes they are found by digging deeply, up to a meter or more, in soil that is moist but not saturated with water (Wake, pers. obs. and Kotharambath, pers. obs., 2017). They have been found by excavating cavities between large trees with root buttresses. Salamanders also have been found at stream edges by working back into sandy soil or gravel.

The most detailed natural history observations of *Oedipina* were ironically recorded for *O. Oedopinola elongatus*, the northernmost species that despite its relatively wide geographic distribution is quite rare. The natural historian and adventurer Sanderson (1941), working in present-day Belize, found specimens in rotten palm fronds and trunks, which he split open to find “sodden fibres” that contained small crabs, insects, whip-scorpions, harvestmen and salamanders, *Oedipina*. The mainly black salamanders moved quickly and disappeared into the stringy mass of rotten palm trunk, but eventually three were captured. He reported (p. 160) “in form they were like worms, being about seven millimeters in diameter throughout, with the tail as fat as the body”. He then described the species accurately and mentioned that the tiny limbs were used for “the push-off”, with further progression by “a furious serpentine wiggle”. He also described the discovery of another individual that apparently had cannibalized two smaller individuals (each about 50 mm head plus body and a 55 mm tail, about 3 mm in diameter). This is a very surprising report because all tropical salamanders feed using a highly projectile tongue to capture typically small insect prey (Deban et al., 2020).

The existence of this complex genus of worm salamanders in Ecuador is enigmatic. Until now the only confirmed record of the genus for Ecuador is a single specimen deposited in the Natural History Museum, London (BMNH 1901.3.29.115) from Imbabura province. Another unconfirmed individual was recorded by Morales-Mite (2004) in Padre Santo, Esmeraldas province (see comments on this specimen below). At last, more than 100 years since first being documented in Ecuador additional specimens of *Oedipina* have been found in the northwestern part of the country lending new insights into the problematic taxonomy of the genus. To our surprise, two of the three Ecuadorian specimens we have examined were found to represent two different species, both of which are described herein.

## Materials & Methods

**Ethics statement.** We conducted this research under permits MAE-DNB-CM-2016-0045 and N° 018-2017-IC-FAU-DNB/MAE, MAE–DNB–CM–2018–0106 and MAE-DNB-CM-2019-0120 issued by the Ministerio del Ambiente del Ecuador. We carried out this research in accordance with the guidelines specified in Reyes-Puig et al. (2019b).

**Taxon sampling.** We examined specimens of *Oedipina* from Ecuador, Colombia and Panama deposited at the following collections: Herpetology Section, Instituto Nacional de Biodiversidad, Quito (DHMECN); Museo de Zoología, Universidad San Francisco de Quito, Quito (ZSFQ), Fundación Herpetológica Gustavo Órces, Quito (FHGO); The Natural History Museum, London (BMNH); Yale Peabody Museum, New Haven (YPM), Museum of Natural History Los Angeles County (LACM) and the Museum of Vertebrate Zoology, Berkeley (MVZ) (Supplementary file I). All museum acronyms follow Sabaj (2016).

For the taxonomic descriptions we used a combination of morphological characters (i.e., external and osteological, as well as tooth counts), genetic divergence and geographic distribution. Similar approaches have been useful to recognize and identify closely related new species of small vertebrates in the northern Andes (Yánez-Muñoz et al., 2018).

The electronic version of this article in Portable Document Format (PDF) will represent a published work according to the International Commission on Zoological Nomenclature (ICZN), and hence the new names contained in the electronic version are effectively published under that Code from the electronic edition alone. This published work and the nomenclatural acts it contains have been registered in ZooBank, the online registration system for the ICZN. The ZooBank LSIDs (Life Science Identifiers) can be resolved and the associated information viewed through any standard web browser by appending the LSID to the prefix http://zoobank.org/. The LSID for this publication is: LSID urn:lsid:zoobank.org:pub:1B4C2057-0FFC-4DAE-9267-240AD991EC7C. The online version of this work is archived and available from the following digital repositories: PeerJ, PubMed Central and CLOCKSS.

**Field work.** Field work was carried out between 29 July and 3 August 2017 in Quinshull near Chical, province of Carchi, Ecuador, during a joint expedition of Universidad San Francisco de Quito USFQ, The Natural History Museum (London), Instituto Nacional de Biodiversidad, and Central University of Kerala; for detailed information see Reyes-Puig et al. (2019a). Herpetological surveys were conducted in foothill forests using visual encounter surveys and actively digging under logs and rocks to search for fossorial amphibians (Heyer et al., 2014). The specimen encountered was photographed alive, euthanized with benzocaine, a sample of muscle tissue was extracted and preserved in 95% ethanol, the individual was fixed in 8% formalin, and preserved in 75% ethanol.

**Figure 1:**
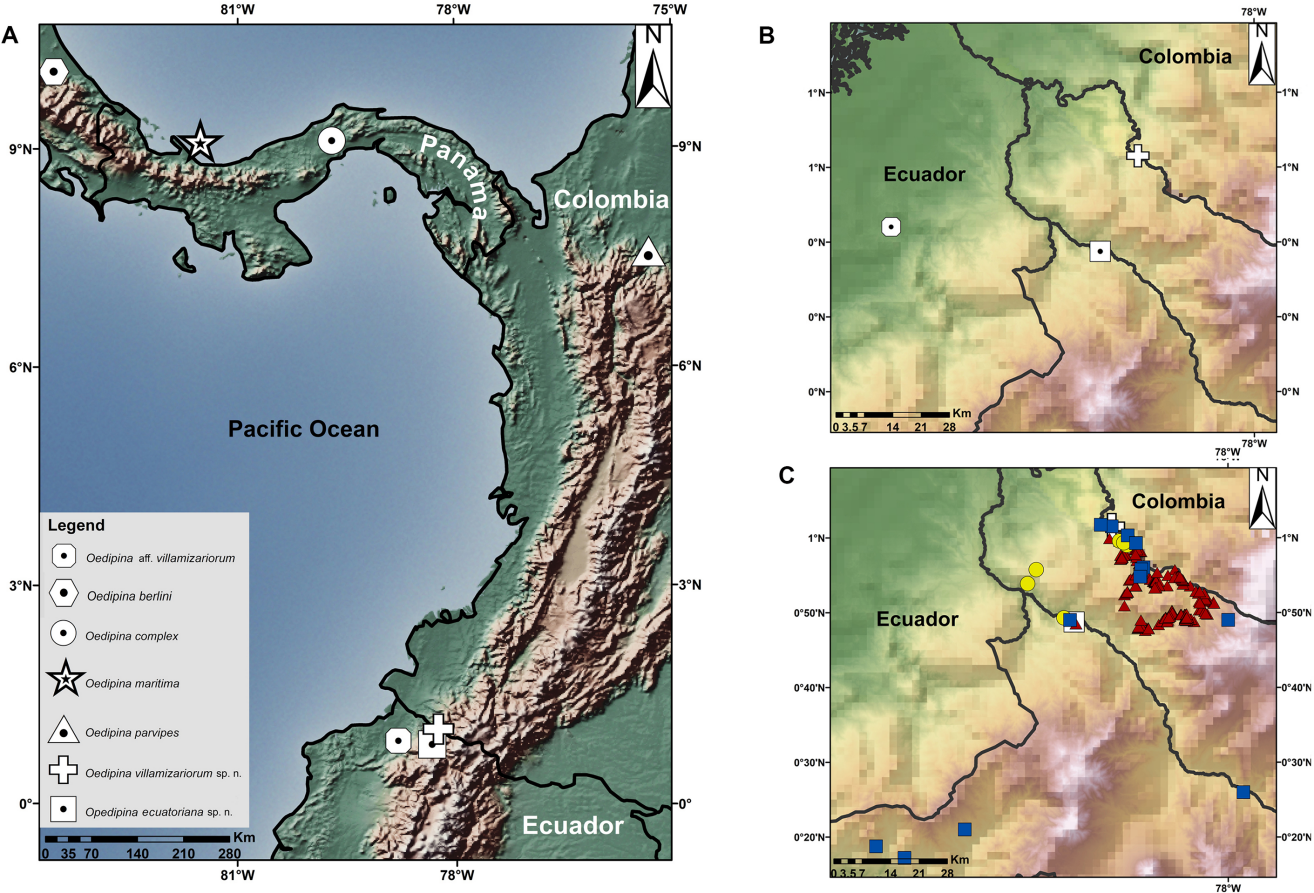
Maps showing the type localities of two new species of *Oedipina* and related species. (A) Type localities of two new species of *Oedipina* and related species, (B) Type localities of three Ecuadorian specimens of *Oedipina*, (C) Sampled points in nearby areas of type localities of *Oedipina* Ecuadorian specimens: Red triangles represent sample points of QCAZ collections, yellow circles represent INABIO collections and blue squares ZSFQ collections.

**Complementary information on collections:** Between 1996–2019 samplings near the type localities, similar ecosystems, and habitats of the two new species of *Oedipina* were conducted. Points sampled within a radius of 60 km of the type localities are shown in Fig. 1, showing amphibian collections represented in Ecuadorian natural history museum collections. Samples were obtained by herpetological surveys conducted under the auspices of the Instituto Nacional de Biodiversidad (INABIO) and Forest protection network of EcoMinga Foundation; Museo de Zoología, Pontificia Universidad Católica del Ecuador (QCAZ) and Museo de Zoología, Universidad San Francisco de Quito (ZSFQ). The accumulated sampling effort invested by QCAZ has been ca. 900 h with 45 persons actively searching herpetofauna in the mentioned period (for more information see Ron, Merino-Viteri & Ortiz, 2020). The INABIO-EcoMinga effort alone involved 20 persons actively searching for herpetofauna for at least 800 h (for more information see Symbiota, 2020). The accumulated sampling effort by ZSFQ has been at least 600 h with 15 persons. The total combined effort produced only a single Oedipina *Oedipina* (described below). Based on the literature (Brame, 1968; Brame & Wake, 1963; Dunn, 1924; Morales-Mite, 2004), known Ecuadorian collections, and this new record, the total specimens of *Oedipina* for Ecuador is three.

**Morphological data.** We followed definitions of morphological characters for *Oedipina* used by Brame (1968) and García-París & Wake (2000). The morphometric measurements were taken with digital calipers by the same person at least three times, and were averaged to the nearest 0.1 mm. The abbreviations of characters are: SL, standard length from snout to posterior margin of vent; TL, tail length; HW, head width; HL, head length; VT, total number of vomerine teeth; PM, total number of premaxillary teeth; MT, total number of maxillary teeth; HW, hand width; FW, foot width; EN, eye to-nostril distance; IN, internarial distance; and EE, interocular distance. The type specimens were examined at the DHMECN and BMNH. The tissue and DNA extraction of the holotype of *Oedipina villamizariorum* sp. n. (DHMECN 14489) was deposited at Museo de Zoología of Pontificia Universidad Católica del Ecuador (QCAZ). Sex was determined following McCranie, Vieites & Wake (2008) and by direct inspection of gonads through a dorsolateral incision.

For the osteological description, the holotype of *Oedipina villamizariorum* sp. n. (DHMECN 14489) was scanned following the protocol described by Reyes-Puig et al. (2019b) for CT scan images analysis. Due to the length of DHMECN 14489, the scan was divided into six sub-scans, each with a duration of about 40–41 min resulting in a total scan duration of 244 min. The exposure time was 615 ms. Three further specimens of *Oedipina* (BMNH 1901.3.29.115 sp. n. from Ecuador, BMNH 1914.5.21.90 *O. parvipes* from Colombia, and BMNH 1929.6.2.39 *O.* cf. *complex* from the Canal Zone, Panama) were scanned at the Natural History Museum, London using both a Nikon Metrology HMX ST 225 scanner at 80 kV and 80 µA or a Zeiss Versa scanner at 50 kV and 80 µA. Osteological terminology follows Darda & Wake (2015), Hanken (1984), Hanken, Wake & Savage (2005), Wake (1966), and Wake et al. (2012). Cartilage structures were omitted from the osteological descriptions, because non-contrast stained micro-CT does not render cartilage.

**Phylogenetic analyses and genetic distances.** We extracted DNA from one specimen of *Oedipina* (DHMECN 14489). The other two specimens (BMNH 1901.3.29.115 and FHGO 9642) were collected in the 19th and 20th century, and genetic samples were not obtained. We extracted DNA from muscle tissue; the DNA sample was preserved in 95% ethanol or RNA later, using standard phenol–chloroform extraction protocols (Sambrook, Fritsch & Maniatis, 1989). We applied a polymerase chain reaction (PCR) to amplify DNA fragments for mitochondrial genes 16S rRNA (primers 16Sar and 16Sbr-H; Kessing et al., 1989) and Cytochrome B (primers MVZ15 and MVZ16; Moritz, Schneider & Wake, 1992). PCR amplification was performed under standard protocols and sequenced by the Macrogen Sequencing Team (Macrogen Inc., Seoul, Korea).

The newly generated DNA sequences are available in GenBank under accession numbers (MT329630 and MT328210). We included GenBank sequences of genes Cytochrome-b,16S rRNA, tRNA^Leu^, ND1, tRNA^Ile^ and tRNA^Gln^, for *Oedipina* and the closely related genera *Bradytriton*, *Cryptotriton*, *Dendrotriton*, *Nototriton*, and *Nyctanolis*. We also included samples of *Bolitoglossa*, *Pseudoeurycea*, and *Thorius* as outgroups to root the tree (based on Rovito et al., 2015). GenBank sequences were originally published by Crawford, Lips & Bermingham (2010), García-París & Wake (2000), Kubicki (2016), Mueller et al. (2004), and Rovito et al. (2015). Preliminary sequence alignment was done with MAFFT 7.2 software with the L-INS-i algorithm (Katoh & Standley, 2013). The alignment was visually examined and manually corrected if needed in MESQUITE version 3.01 (Maddison & Maddison, 2014). The aligned matrix is available at Zenodo.org: https://doi.org/10.5281/zenodo.3628754. The final DNA matrix had 2,276 bp and 96 terminals.

Phylogenetic trees were obtained using maximum likelihood searches with software IQ-TREE version 1.6.8 (Nguyen et al., 2015). We partitioned the matrix by gene and codon position to find the best model of evolution for each partition. To accomplish that, we used the command MFP (Chernomor, Von Haeseler & Minh, 2016; Kalyaanamoorthy et al., 2017) in software IQ-TREE 1.6.8. To find the best phylogeny we ran IQ-TREE 1.6.8 under the commands “-m MFP -b 200 -alrt 1000”. Branch support was assessed by two criteria: (1) non-parametric bootstrap (200 replicates) and (2) Shimodaira-Hasegawa Like (SHL) approximate Likelihood-Ratio Test (aLRT), 1,000 replicates (Guindon & Gascuel, 2003)

## Results

**Figure 2:**
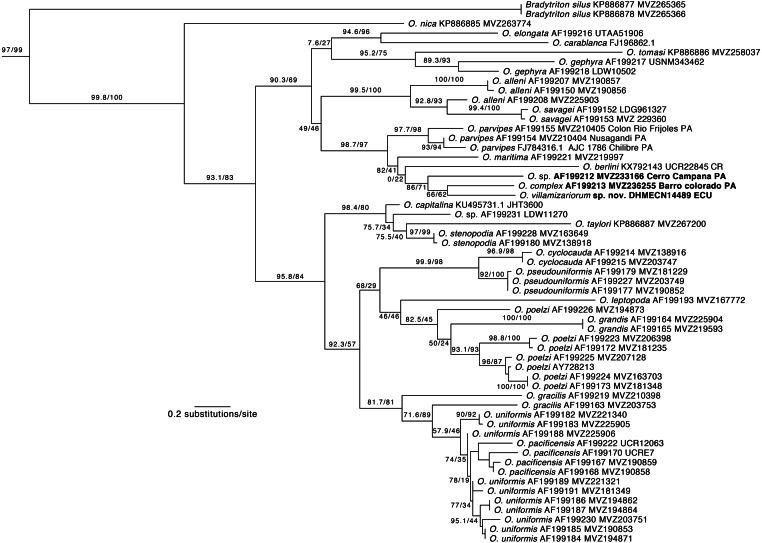
Phylogeny of *Oedipina*. Maximum likelihood tree inferred from a partitioned analysis of 2,276 aligned sites of Cytochrome-b,16S rRNA, tRNA^Leu^, ND1, tRNA^Ile^ and tRNA^Gln^ showing phylogenetic relationships of *Oedipina*. SH-aLRT support (before slash) and non-parametric bootstrap support (after slash) are shown as percentages on branches. SH-aLRT values above or equal to 80 are interpreted as strong support. The outgroup is not shown. GenBank accession number for one of the genes and voucher collection number are shown after the species name; locality is shown for samples closely related to the new species. Abbreviations: CR, Costa Rica; ECU, Ecuador; PAN, Panamá.

**Phylogenetic relationships.** Our phylogeny (Fig. 2) shows strong support for the monophyly of *Oedipina* and a sister relationship with *Bradytriton*. Within *Oedipina*, we found strong support for a clade that unites *O. berlini*, *O. complex*, *O, maritima*, and *O. parvipes*. The sample from Chical, Carchi (DHMECN 14489) is the sister taxon of a sample of *O. complex* from Barro Colorado (Panama). There are two samples of *Oedipina complex*, one from Cerro Campana (Panama) and another from Barro Colorado that is ∼15 km from the type locality of *O. complex* and likely represents *O. complex* sensu stricto. The sample from Cerro Campana may represent a different species (García-París & Wake, 2000) and is best called *Oedipina* sp.. We also found paraphyly among samples of *O. alleni*, *O. pacificensis*, *O. poelzi*, and *O. uniformis,* suggesting either cryptic diversity or misidentified specimens. The uncorrected p-genetic distance between *O. complex* from Barro Colorado and the Chical, Carchi (DHMECN 14489) specimen is 2.7% along 534 bp of the 16S gene. The 16S genetic distance between *O. complex* from Cerro Campana and Chical is 4.3%.

**Figure 3:**
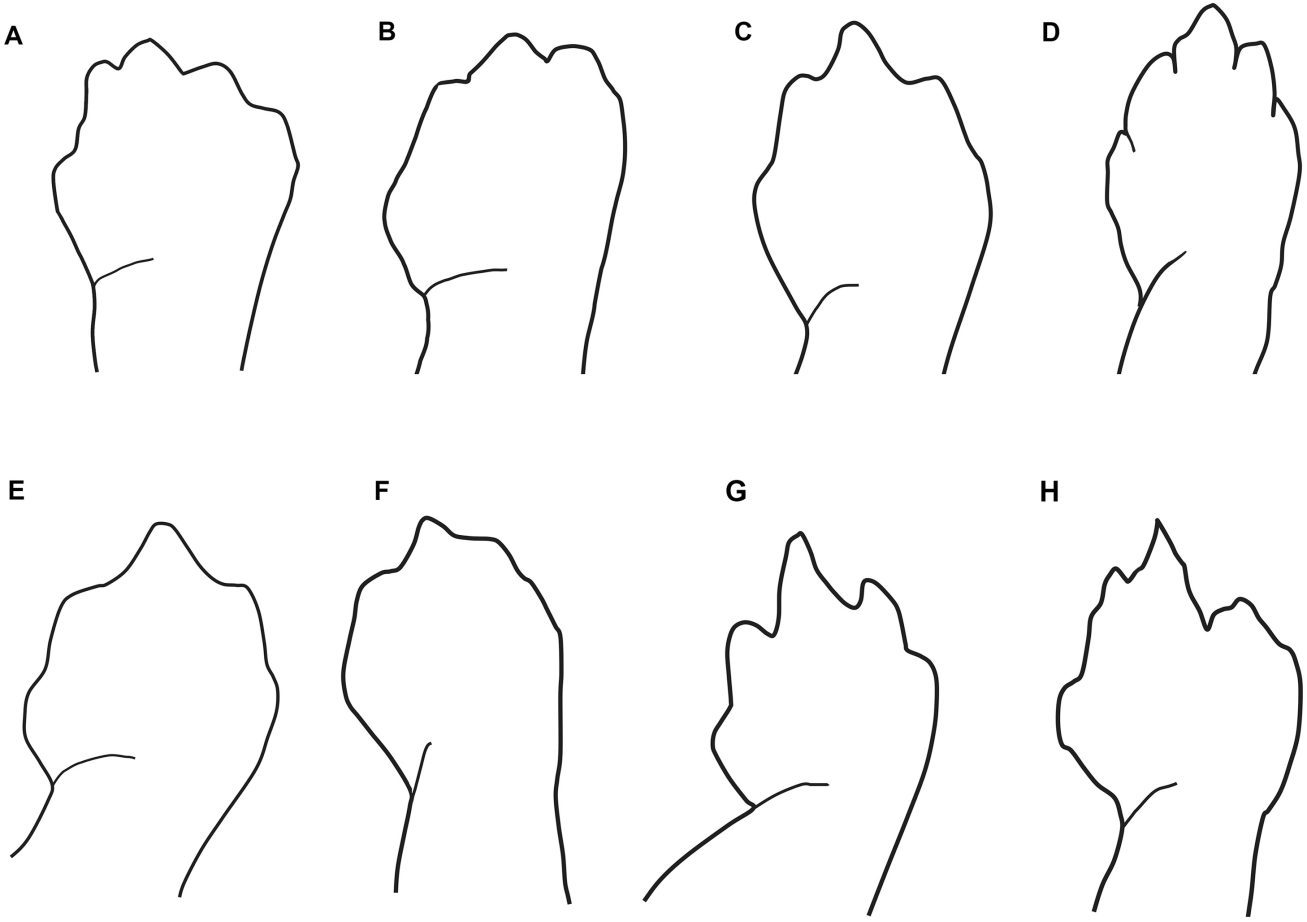
Outlines of the shape of the right foot for samples of eight species of *Oedipina* (*Oedopinola*). (A) *O. villamizariorum* sp. n., DHMECN 14489 (42.1 SL), holotype, Quinshull, Carchi, Ecuador; (B) *O. ecuatoriana* sp. n., BMNH 1901.3.29.115 (45.6 SL), holotype, Paramba, Imbabura, Ecuador; (C) *O*. aff. *villamizariorum*, FHGO 9642, (39.8 SL), Padre Santo, Esmeraldas, Ecuador; (D) *O*. sp. MVZ 233166 (37.2 SL), Cerro Campana, Panamá; (E) *O. berlini*, holotype, UCR 22845 (38.7 SL) Costa Rican Amphibian Research Center’s Guayacán Rainforest Reserve, Costa Rica; (F) *O. complex* MVZ-DBW 5105 (35.0 SL), Peninsula Bohío, Prov. Colón, Panamá; (G) *O. maritima* USNM 529981 (44.3 SL), holotype; (H) *O. parvipes* LACM 134872 (53.9 SL), Barro Colorado Island, Panamá. (E extracted from Kubicki, 2016 and D, F–H extracted from García-París & Wake, 2000, with permission).

**Figure 4:**
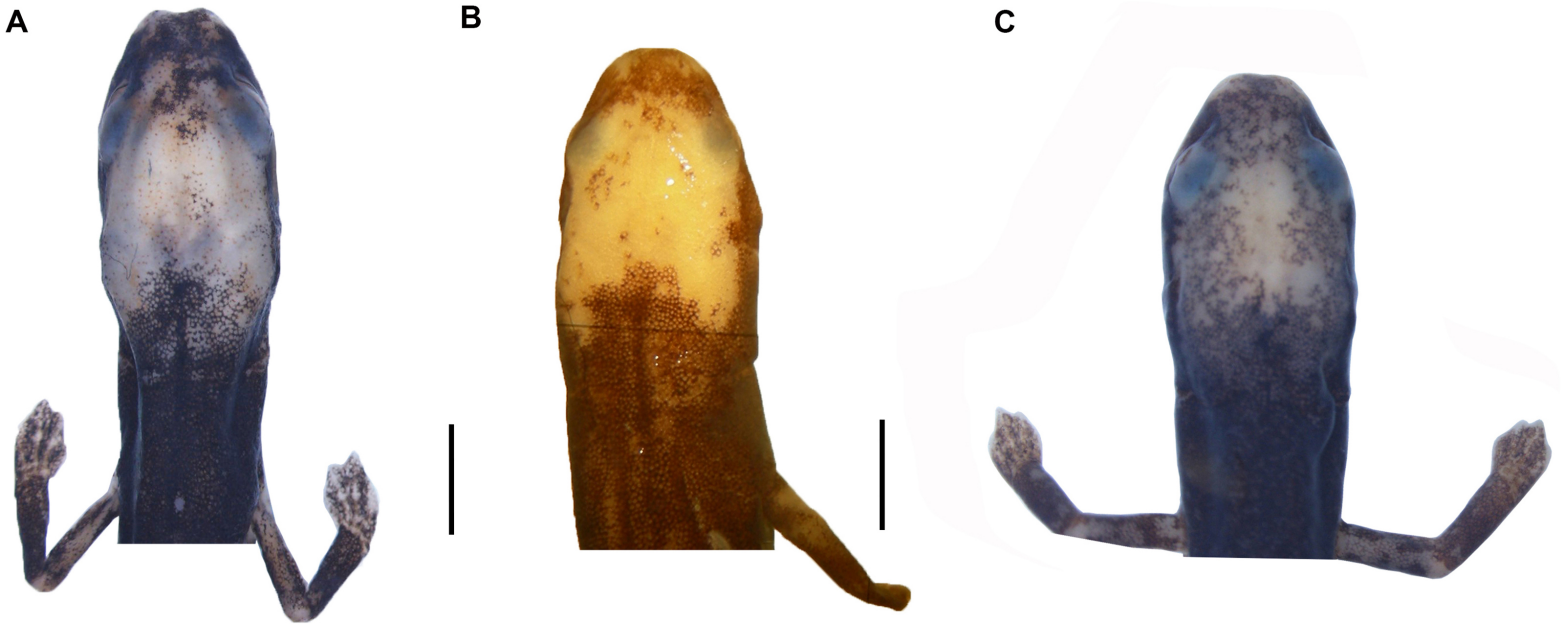
Head detail of (A) *Oedipina villamizariorum* sp. n. DHMECN 14489 (42.1 SL), holotype; (B) *O. ecuatoriana* sp. n. BMNH 1901.3.29.115 (45.6 SL) holotype; (C) *O*. aff. *villamizariorum* FHGO (39.8 SL). Scale bar represent 2 mm.

**Figure 5:**
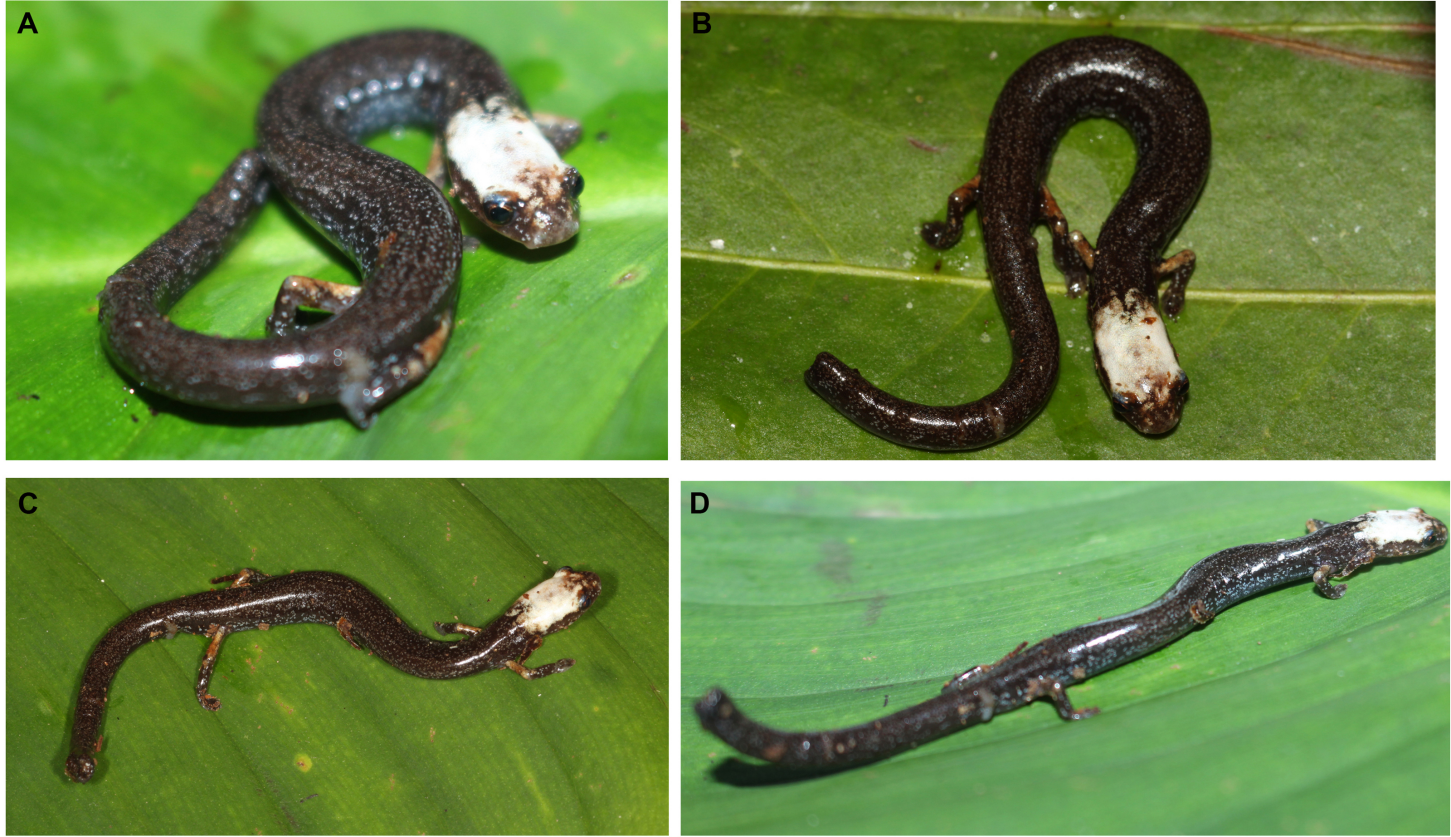
Life pattern coloration of *Oedipina villamizariorum* sp. n. (holotype, DHMECN 14489, SL = 42.1 mm). (A–B) frontal and dorsal view; (C–D) dorsal and lateral view. Photographs by Carolina Reyes-Puig (A, D) and Gabriela Bittencourt-Silva (B, C).

**Figure 6:**
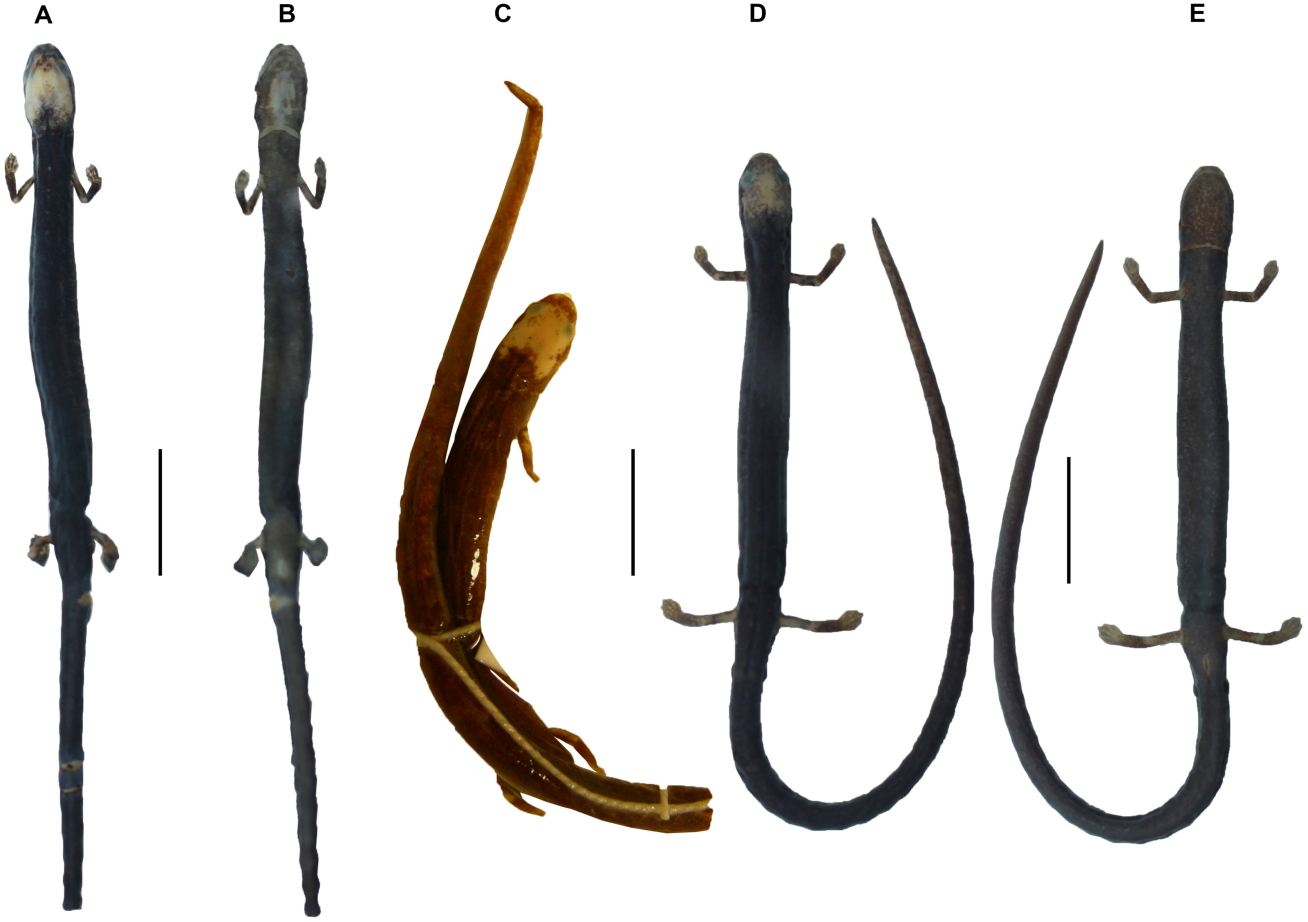
Dorsal and ventral view of (A–B) *Oedipina villamizariorum* sp. n. DHMECN 14489 (42.1 mm SL), holotype; dorsal view of (C) *O. ecuatoriana* sp. n. BMNH 1901.3.29.115 (45.6 mm SL) holotype; dorsal and ventral view of (D–E) *O*. aff. *villamizariorum* FHGO (39.8 mm SL). Scale bar represents 10 mm.

**Figure 7:**
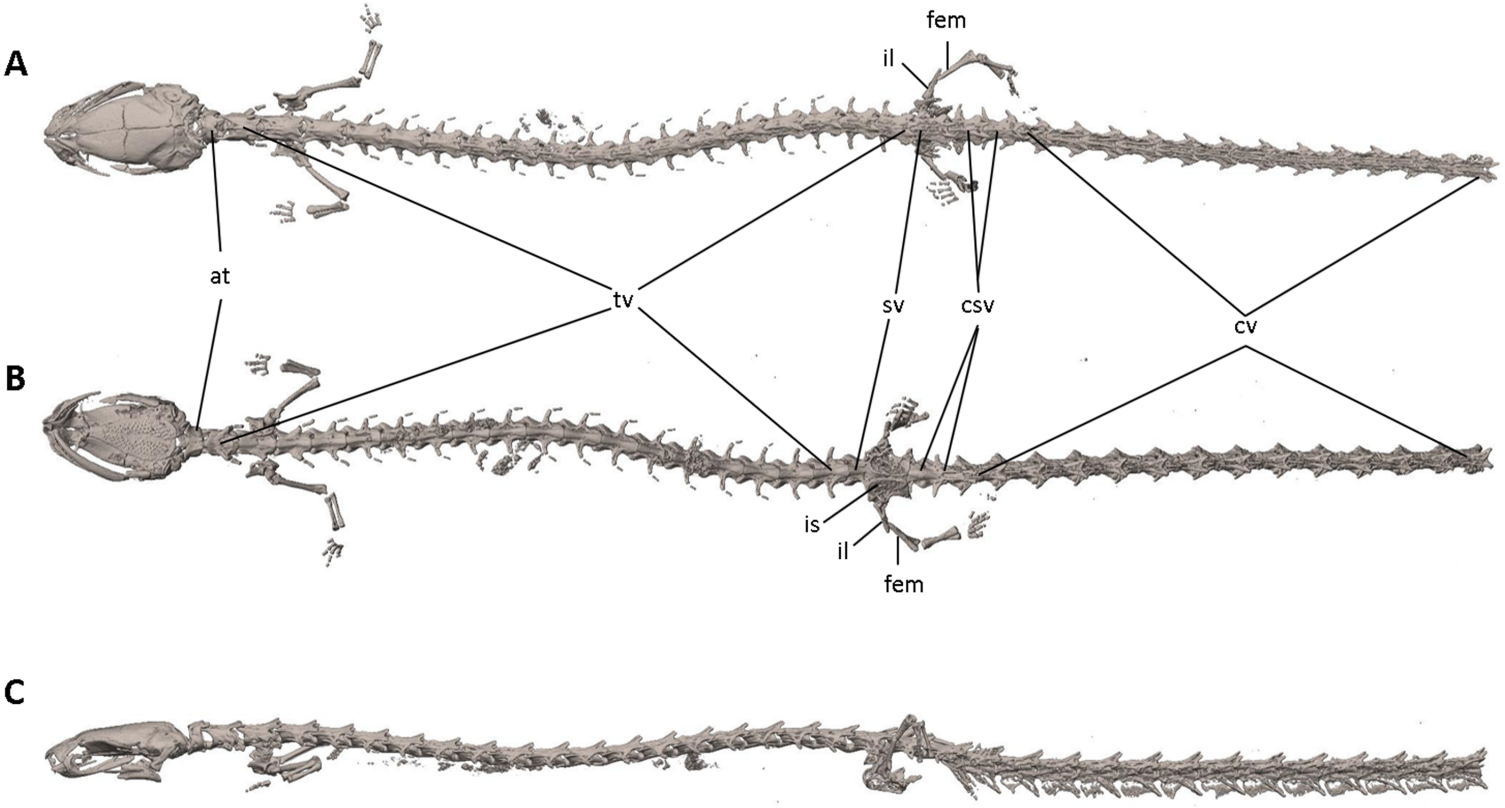
Osteology of *Oedipina villamizariorum* sp. n. (holotype, DHMECN 14489, SL =42.1 mm). The full skeleton is shown in (A) dorsal, (B) ventral, and (C) lateral views. at, atlas; cv, caudal vertebrae; csv, caudosacral vertebrae; fem, femur; il, ilium; is, ischium; sv, sacral vertebra; tv, trunk vertebrae.

**Figure 8:**
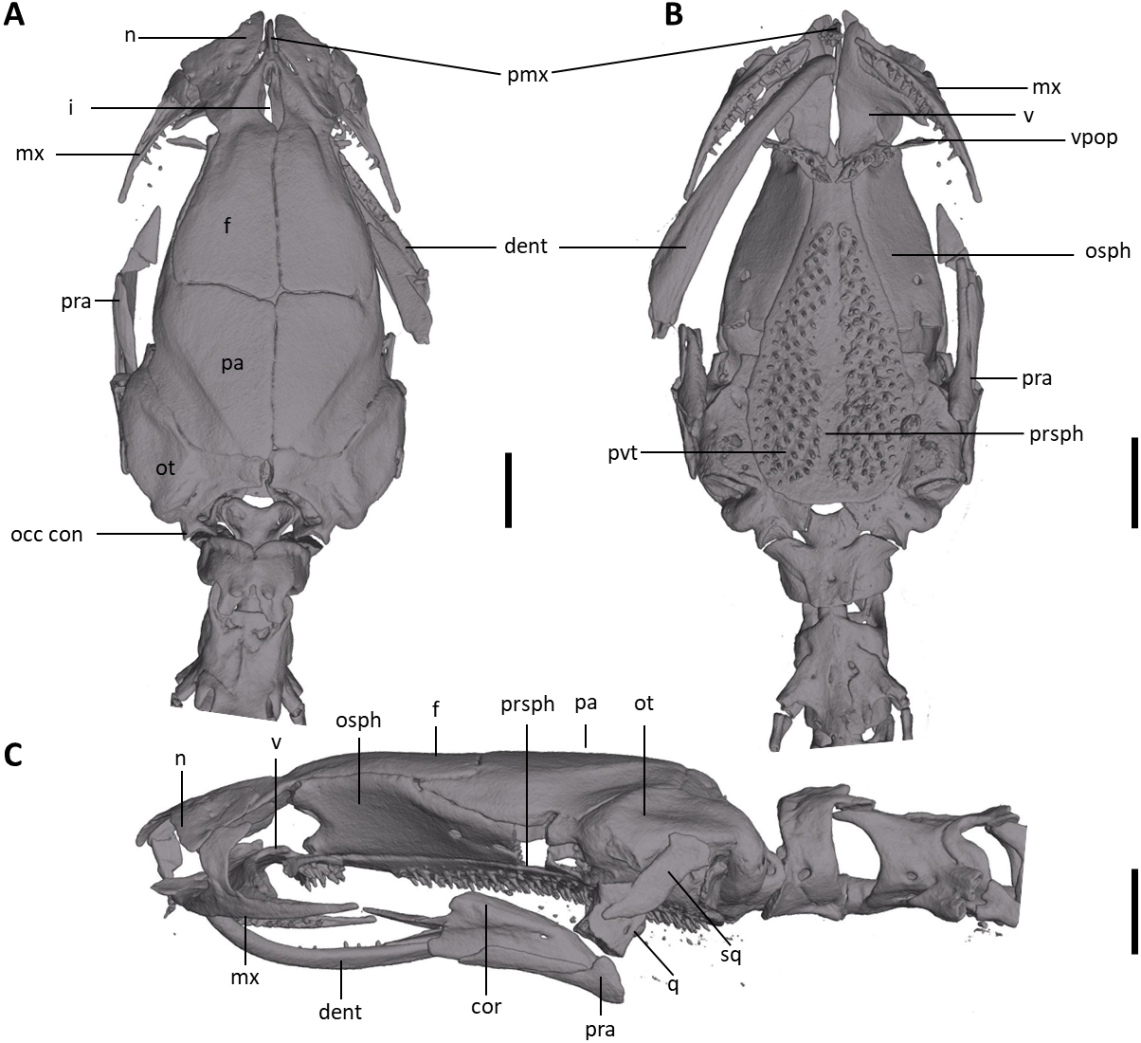
Skull of *Oedipina villamizariorum* sp. n. (holotype, DHMECN 14489). Shown in (A) dorsal, (B) ventral, (C) lateral views. cor, coronoid process of prearticular; dent, dentary; f, frontal; i, internasal fontanelle; mx, maxilla; n, nasal; occ con, occipital condyle; osph, orbitosphenoid; ot, otic-occipital; pa, parietal; pmx, premaxilla; pra, prearticular; prsph, parasphenoid; pvt, posterior vomerine teeth; q, quadrate; sq, squamosal; v, vomers; vpop, vomer preorbital process. Scale bars = 1 mm.

**Figure 9:**
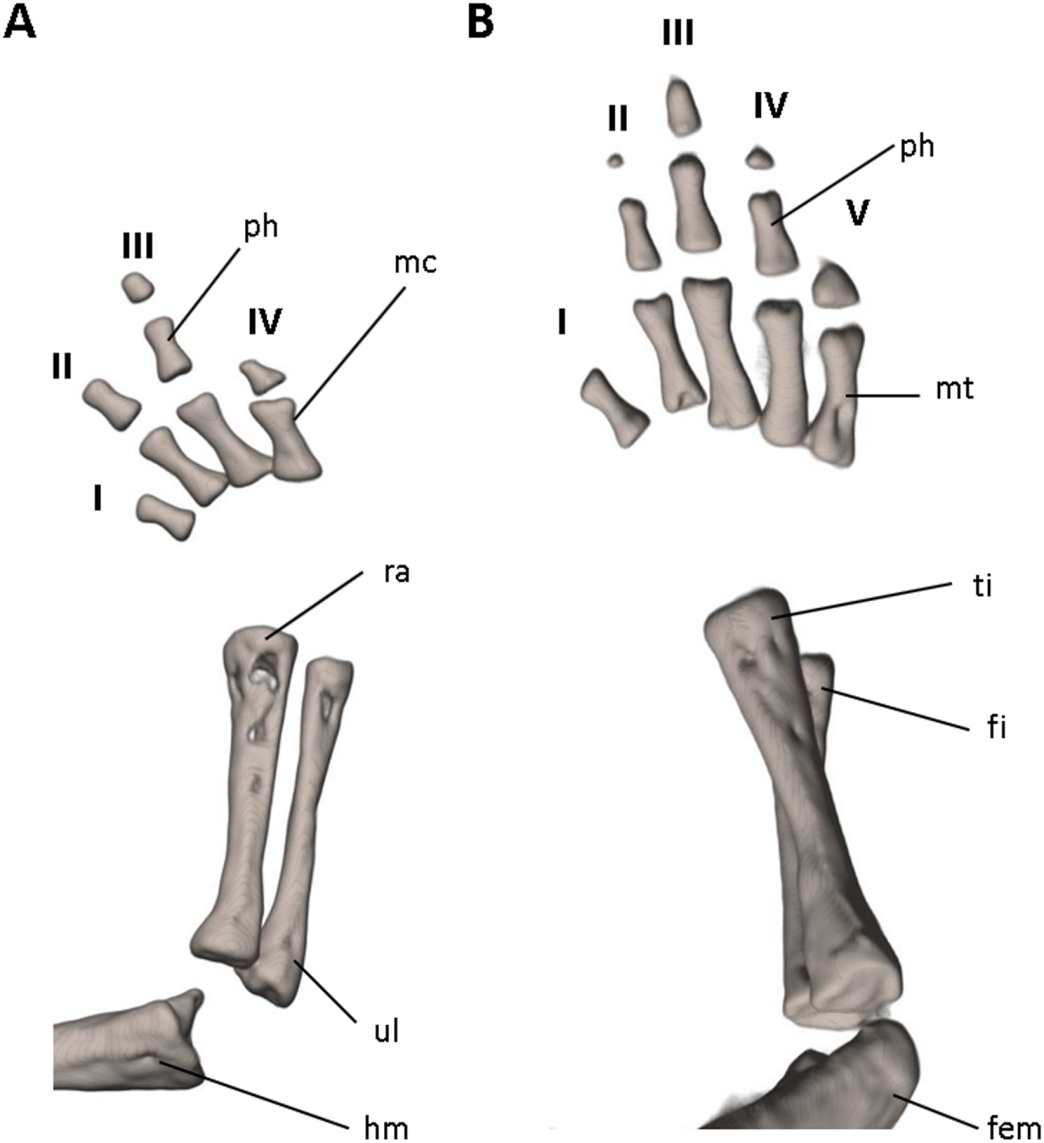
Osteology of the limbs of *Oedipina villamizariorum* sp. n. (holotype, DHMECN 14489, SL =42.1 mm). The (A) right forelimb and (B) the right foot are shown in dorsal aspects. Digits numbered I–V. fem, femur; fi, fibula; hm, humerus; mc, metacarpalia; mt, metatarsalia; ph, phalanges; ra, radius; ti, tibia; ul, ulna.

### Systematic accounts

***Oedipina villamizariorum* sp. n.**

Figs. 1–9

LSID urn:lsid:zoobank.org:act:A47B831D-EF23-44FC-929D-E7575A50F1DF

**Proposed standard English name:** Villamizar’s worm salamander

**Proposed standard Spanish name:** Salamandra gusano de Villamizar

**Holotype.**—DHMECN 14489 (Field ID, MW 9902), female, from Quinshull, Chical, provincia de Carchi, Ecuador (1.02525, −78.2575277, 921 m) (Fig. 1), collected 02 August 2017 by Ramachandran Kotharambath, Mark Wilkinson, María Torres-Sánchez and Francesca Angiolani.

**Diagnosis.** We assign *Oedipina villamizariorum* to the genus *Oedipina* (Keferstein, 1868) based on the characterization of Brame (1968): 18 trunk vertebrae (18–23 in the genus, versus 14 in all other Bolitoglossini); 17 costal grooves per side; sublingual fold present; body greatly attenuated; limbs very short (8.5 costal folds exposed when limbs appressed to sides of trunk); very long tail, up to more than twice standard length (the tail is broken but based on the size of remaining vertebrae apparently was very long when complete). In addition, we assign the new species to the subgenus *Oedopinola* (Hilton, 1946) (equivalent to the *parvipes* group of Brame, 1968) based on the characterization of Brame (1968) and García-París & Wake (2000) of having 18 trunk vertebrae and often possessing a white face mask (i.e., dorsal coloration on the head).

*Oedipina villamizariorum* is a medium-sized member of the genus (SL 42.1 mm) with a narrow, relatively pointed head with a blunt snout. The new species differs from *O. parvipes* (topotype) by having a somewhat more blunt-tipped snout; 26 maxillary teeth (19 in the holotype but only 2 in the topotypic individuals of *O. parvipes*); dorsolaterally oriented eyes that are moderate in size (small in *O. parvipes*); From *O. complex* sensu stricto by having a longer and more pointed head with a blunt snout (short and rounded to blunt tipped snout), limb interval $8 \frac{1}{2} $ (7 −$8 \frac{1}{2} $); tip of the digits pointed and moderately long (blunt and short). *Oedipina villamizariorum* also can be distinguished from *O. maritima* (characters in parentheses) by having pointed and blunt-tipped snout (pointed and elongate); moderate in size dorsolaterally oriented eyes (small laterally orientated); 26 maxillary teeth (0–8). The new species differs from *O. elongata* and *O. carablanca* by smaller size (both exceed 50 mm SL) and the presence of maxillary teeth. *Oedipina villamizariorum* can be distinguished from *O. berlini* (characters in parentheses) by a narrow, relatively pointed head with a blunt snout and dorsolaterally oriented eyes that are moderate in size (flat head, more than twice as wide as high, with relatively large protruding eyes), the dorsum is chocolate, densely dotted with light brown, turquoise cream and brownish orange and with a white face mask on the dorsal surface of the head (colored with a mixture of pale earthy tones ranging from tan to dark reddish brown, with finer white and dark brown to black spots and irregular markings scattered throughout). The new species differs from *O. fortunensis* by having a narrow, relatively pointed head with a blunt snout (a narrow head and a relatively short, rounded snout in *O. fortunensis*), and dorsum chocolate in color, densely dotted with light brown, turquoise cream and brownish orange (brown with white flecks in *O. fortunensis*). *Oedipina villamizariorum* differs from *O. ecuatoriana* sp. n., described below, by its apparently smaller size (the unique specimen (holotype) of *O. ecuatoriana* is a male, typically the smaller of the two sexes, yet it is larger than the female holotype of *O. villamizariorum*), its narrower and more pointed snout, its pointed rather than round-tipped long digits, and much less white pigmentation on the head. The two species differ in several osteological characters (see below).

**Description of holotype (Figs. 3–6)**. A slender species, moderate in size; adult female (SL 42.1 mm). The head is narrow, cylindrical and pointed with blunt-tipped snout; SL 10 times head width. SL 5.7 times head length. Nostrils are evident and slightly laterally directed. Nasolabial protuberances are short (evident and swollen in life). The snout extends slightly over the underslung lower jaw. Eyes are moderate in size and dorsolaterally oriented; eyes extend beyond the lateral margins of the head (less evident in the preserved specimen). The suborbital groove does not intersect the lip line. There are no premaxillary teeth. Maxillary teeth (26) are evident, slightly elongated and sharp. Vomerine teeth (14) are conspicuous, borne in a long, V-shaped row. There are 17 costal grooves between the limbs, counting one each in the axilla and the groin (18 trunk vertebrae). Limbs are relative short and slender; limb interval is 8.5. Hands and feet are small and narrow. The digits are syndactylous, with tips of the digits pointed; tips of toes 2-3-4 (central digits) are free. The free tips are slender and sharply pointed. Fingers, in order of decreasing length, are 3-2-4-1; toes 3-4-2-5-1. The tail is round, cylindrical, narrow in cross-section and relatively long; the specimen has lost the distal portion of the tail.

**Measurements of holotype (in mm).—**Head width 4.2; snout to gular fold (head length) 7.5; head depth at posterior angle of jaw 2.4; eyelid width 0.8, eyelid length 1.7; eye to nostril 1.4; anterior rim of orbit to snout 1.8; horizontal orbit diameter 1.6; interorbital distance 2.2; distance separating eyelids 1.7; nostril diameter 0.3; snout projection beyond mandible 0.5; distance from eye to postorbital groove 2.3; snout to posterior angle of vent (standard length) 42.1; snout to anterior angle of vent 41; snout to forelimb 10.2; axilla to groin 27.8; limb interval 8.5; shoulder width 3; tail length 26.3; tail width at base 3.2; tail depth at base 3.9; forelimb length (to tip of longest digit) 6.6; hind-limb length 7.3; hand width 1.1; foot width 1.9; free length of longest toe 0.4. Numbers of teeth: premaxillary 0; maxillary 26; vomerine 14.

**Coloration in life of the holotype (Fig. 5).** Dorsal surfaces chocolate, densely dotted with light brown, turquoise cream and brownish orange. A white face mask (described below), bounded with brownish orange. The upper eyelid slightly pigmented with turquoise. Loreal and rostral region with white scattered marks. Flanks and dorsal surfaces of hand and feet with distinctive irregular turquoise stains. Dorsal surfaces of humerus and femur orange. Ventral surfaces of the body brownish cream. Iris coppery brown.

**Coloration of the holotype in alcohol (Fig. 6).** Dorsum and flanks grayish brown, with white dots scattered throughout the vertebral and costal region. A white face mask that covers the frontal, parietal, occipital and postoccipital regions; slightly extending to the nuchal fold; without extending to the temporal, subocular and nasal regions. Costal grooves on the body and tail are dirty white. Dorsal surfaces of arm and leg orange. Ventral surfaces of the body and throat are densely dotted with cream between black interspaces; there are two clearly depigmented portions, a collar-like form delimited with brown, and a triangular form in the middle portion of the throat. Ventral surfaces of the fore- and hind limbs insertions depigmented. Palmar and plantar surfaces are translucent.

**Osteology.** The following account is based on the female holotype of *Oedipina villamizariorum* (DHMECN 14489), the only known specimen of the new species (Figs. 7–9). The skull is robust and well ossified. The premaxilla is small and single and is articulated to each maxillary bone. The frontal processes of the premaxilla are only very slightly divided and form the extreme anterior margin of the internasal fontanelle, which is otherwise encircled by the anterior projections of the frontals. Prefrontals and septomaxillae are absent and the nasals and large maxillary facial lobes occupy the vacated area. The maxillae terminate at approximately the center of the orbit. There are 12 teeth on the left maxilla (right maxilla is partially covered by the dentary bone). The facial lobe of the maxilla is almost completely anterior to the midpoint of toothed portion and strongly articulates with the lateral margin of the nasal. Nasals are more or less triangular in dorsal view and articulate firmly with the anterior projections of the frontals. They are not in median contact and do not overlap the frontal process of the premaxilla. Frontoparietal fontanelle absent. Frontals are broad posteriorly and taper towards the internasal fontanelle. They are well articulated to one another medially, to frontal processes of the premaxilla anteriorly, to nasals anterolaterally, to underlying orbitosphenoids laterally, and to parietals posteriorly. The posterolateral boarder of each parietal contributes to a low ridge on the otic-occipitals. With the frontals they form an almost straight suture. Posteriorly frontals do not reach the posterior margins of the orbitosphenoids. The frontals have an anteromedial spinous projection that extends besides the premaxillary processes. The frontals and maxillary facial lobes are not in contact. The parietals are more or less rectangular and have a posterolateral depression. They are well articulated to one another medially, to orbitosphenoids laterally, to otic-occipitals posteriorly, and to frontals anteriorly. The squamosal is oblique and articulates firmly with the otic-occipital and the quadrate; the rudimentary spur on the posterior border of the squamosal is very reduced but visible. The quadrate is massive. The operculum is a simple, round disk and lacks a stylus. The occipital condyles are short and stout. The paired vomers are large and well developed and are separated along most of their midline to form a narrow but long fontanelle. The bones approach one another in front of the fontanelle. They articulate anteriorly with the maxillae, posterodorsally with the orbitosphenoid, and posteriorly with the parasphenoid. The preorbital processes of the vomers are relatively long and slender and extend laterally beyond lateral margins of body of vomers and approach the maxillae. Vomer bears 14 teeth along its posterior margin. About 50–60 articulated teeth are present in each broad posterior vomerine patch. Both patches are distinctly separated along the midline. The orbitosphenoids are well developed and articulate dorsally with the frontals and parietals, ventrally with the parasphenoid, and anteroventrally with the vomers. The optic fenestrae are enclosed in bone and are located in the posterior one third of the bone. The parasphenoid is long but does not reach the anterior margin of the orbitosphenoid. It is oval-shaped posteriorly and medially, and extended anteriorly, where it slightly narrows towards the tips. Anteriorly the parasphenoid overlaps with the vomers, it strongly articulates with the orbitosphenoids laterally, and with the otic-occipitals laterally and posteriorly. The lower jaw is broken on both sides and the left dentary is lacking. The right dentary is well developed and bears a series of 9 teeth. The prearticular is relatively large and robust and envelopes the apparently mineralized articular cartilage.

Vertebral column with 1 atlas, 18 trunk, 1 sacral, 2 caudosacral, and 13 caudal vertebrae. The atlas is fully ossified and distinctly shorter than the trunk vertebrae. The ribs are very short. The last two trunk vertebrae lack ribs. The caudosacral vertebra and the first two caudal vertebrae have a single median dorsal crest. Commencing on the third caudal vertebrae the crests divide and form double, parallel crests on either side of the midline. Transverse processes are present on all caudal vertebrae. A well-developed keel is present on hemal arch of all caudal vertebrae, but no keel on hemal arch of caudosacral vertebra.

**Figure 10:**
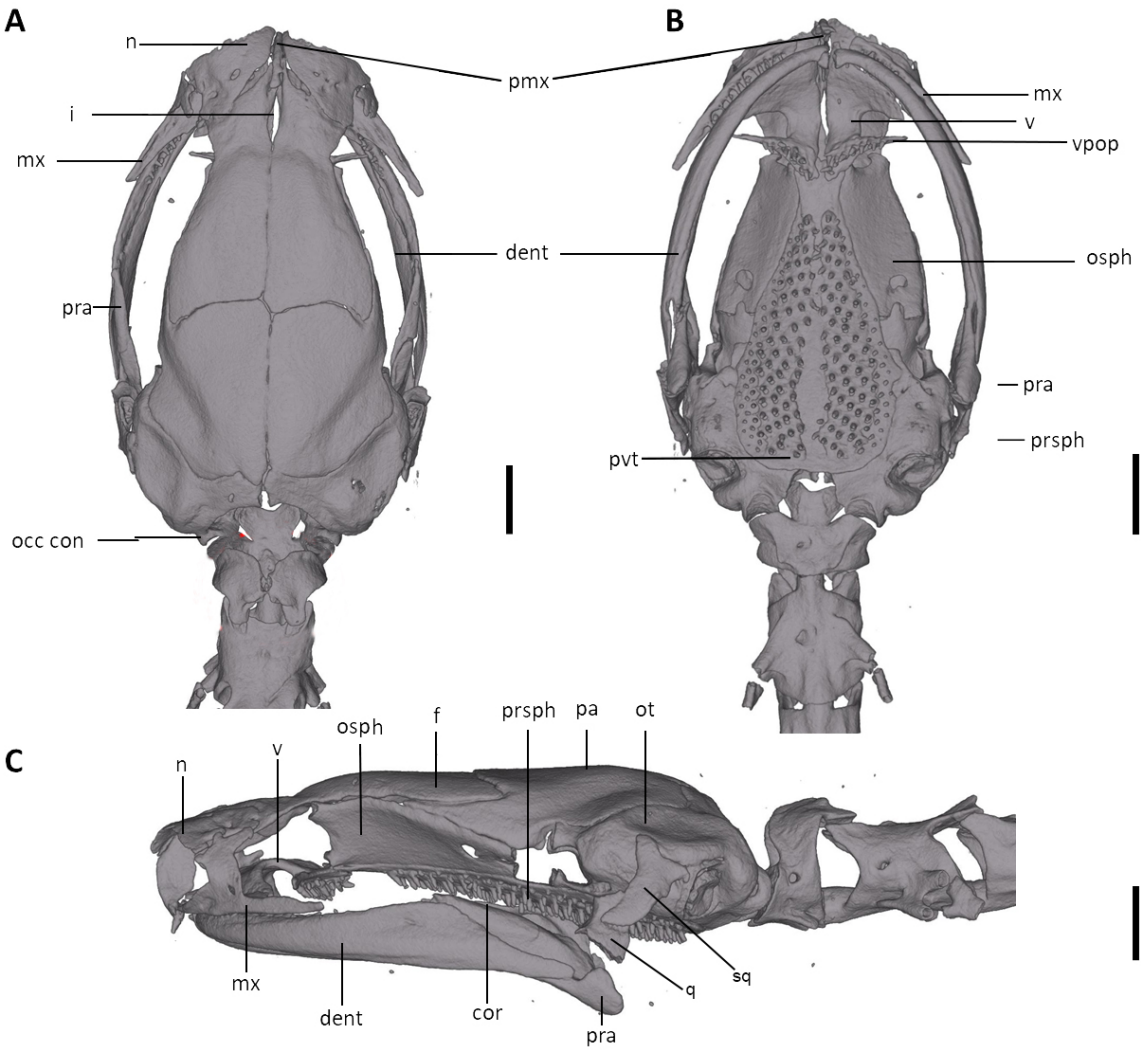
Skull of *Oedipina* aff. *villamizariorum* (FHGO 9642). Shown in (A) dorsal, (B) ventral, (C) lateral views. cor, coronoid process of prearticular; dent, dentary; f, frontal; i, internasal fontanelle; mx, maxilla; n, nasal; occ con, occipital condyle; osph, orbitosphenoid; ot, otic-occipital; pa, parietal; pmx, premaxilla; pra, prearticular; prsph, parasphenoid; pvt, posterior vomerine teeth; q, quadrate; sq, squamosal; v, vomers; vpop, vomer preorbital process. Scale bars = 1 mm.

Limbs moderately developed. No tibial spur visible. The mesopodial elements are unmineralized. Four fingers and five toes are present. The phalangeal formula is 0-1-2-1 for each hand and 0-2-2-2-1 for each foot. The last phalanx of the second toe is very short and spherical. For comments on osteological characters in *Oedipina* aff. *villamizariorum* (Fig. 10) see Remarks section.

**Figure 11:**
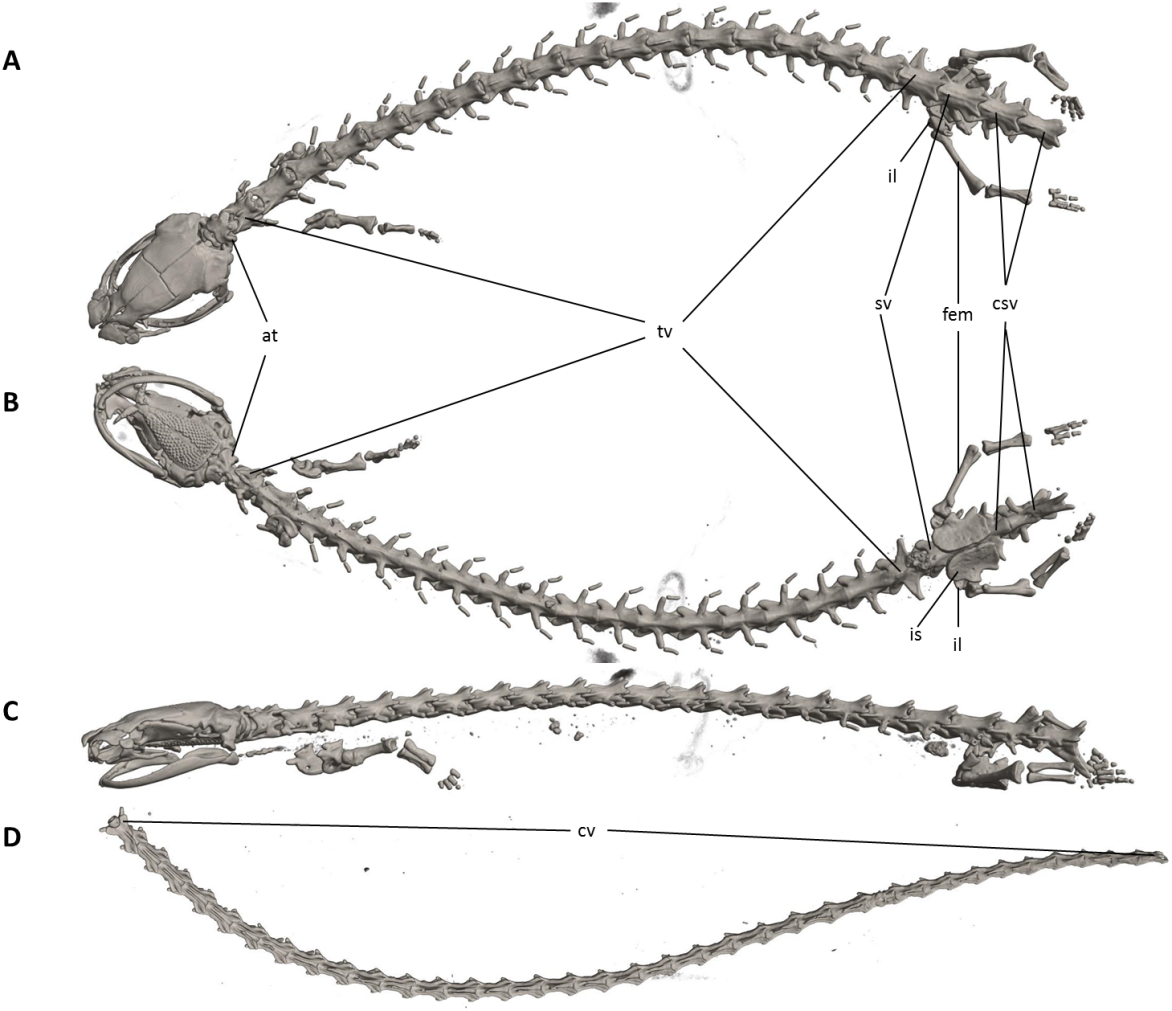
Osteology of *Oedipina ecuatoriana* sp. n. (holotype, BMNH 1901.3.29.115, SL =45.6 mm). The full skeleton is shown in (A) dorsal, (B) ventral, and (C) lateral views, and (D) dorsal view of tail. at, atlas; cv, caudal vertebrae; csv, caudosacral vertebrae; fem, femur; il, ilium; is, ischium; sv, sacral vertebra; tv, trunk vertebrae.

**Figure 12:**
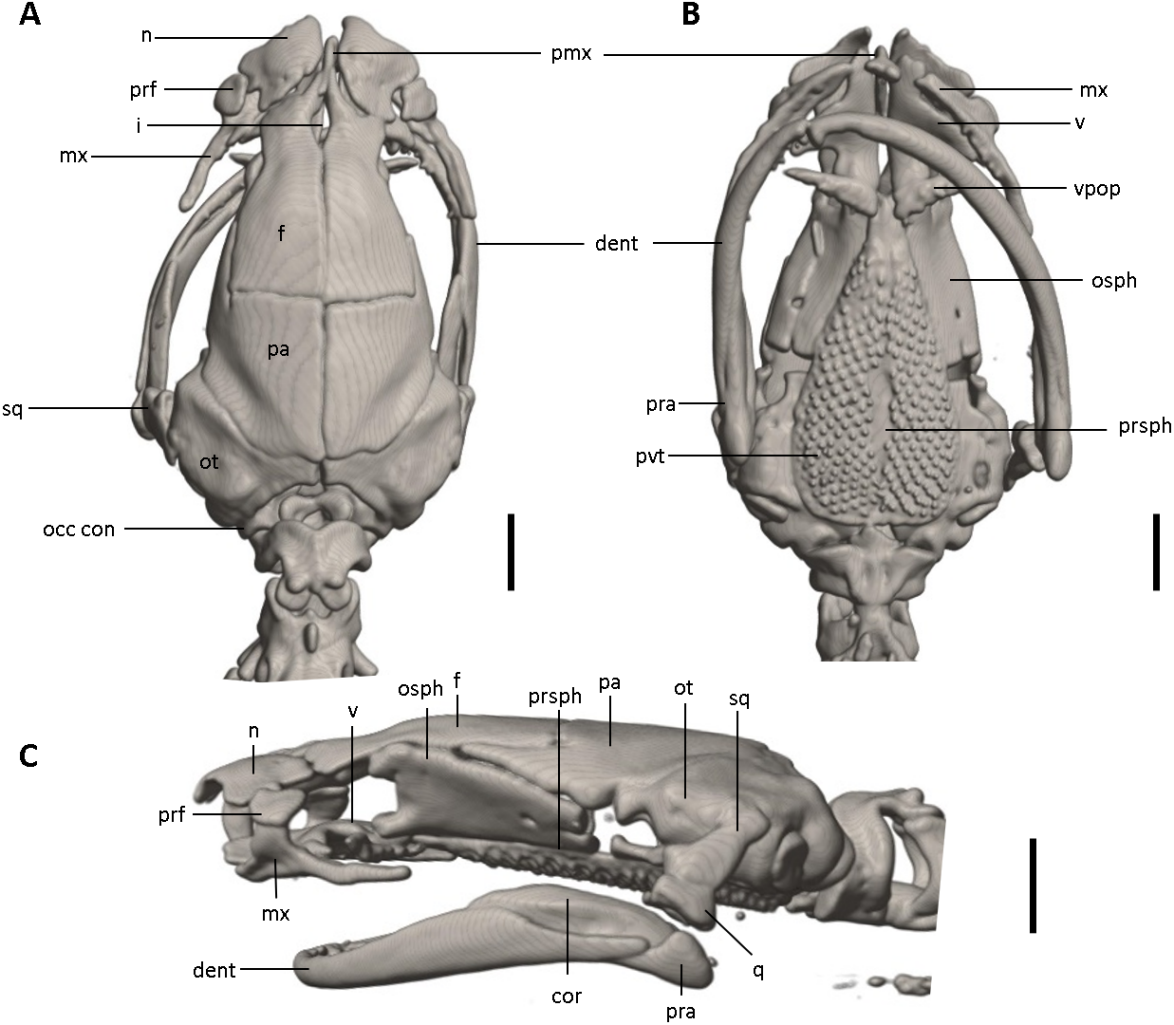
Skull of *Oedipina ecuatoriana* sp. n. (holotype, BMNH 1901.3.29.115). Shown in (A) dorsal, (B) ventral, (C) lateral views. cor, coronoid process of prearticular, dent, dentary; f, frontal; i, internasal fontanelle; mx, maxilla; n, nasal; occ con, occipital condyle; osph, orbitosphenoid; ot, otic-occipital; pa, parietal; pmx, premaxilla; pra, prearticular; prf, prefrontal; prsph, parasphenoid; pvt, posterior vomerine teeth; q, quadrate; sq, squamosal; v, vomers; vpop, vomer preorbital process. Scale bars = 1 mm.

**Figure 13:**
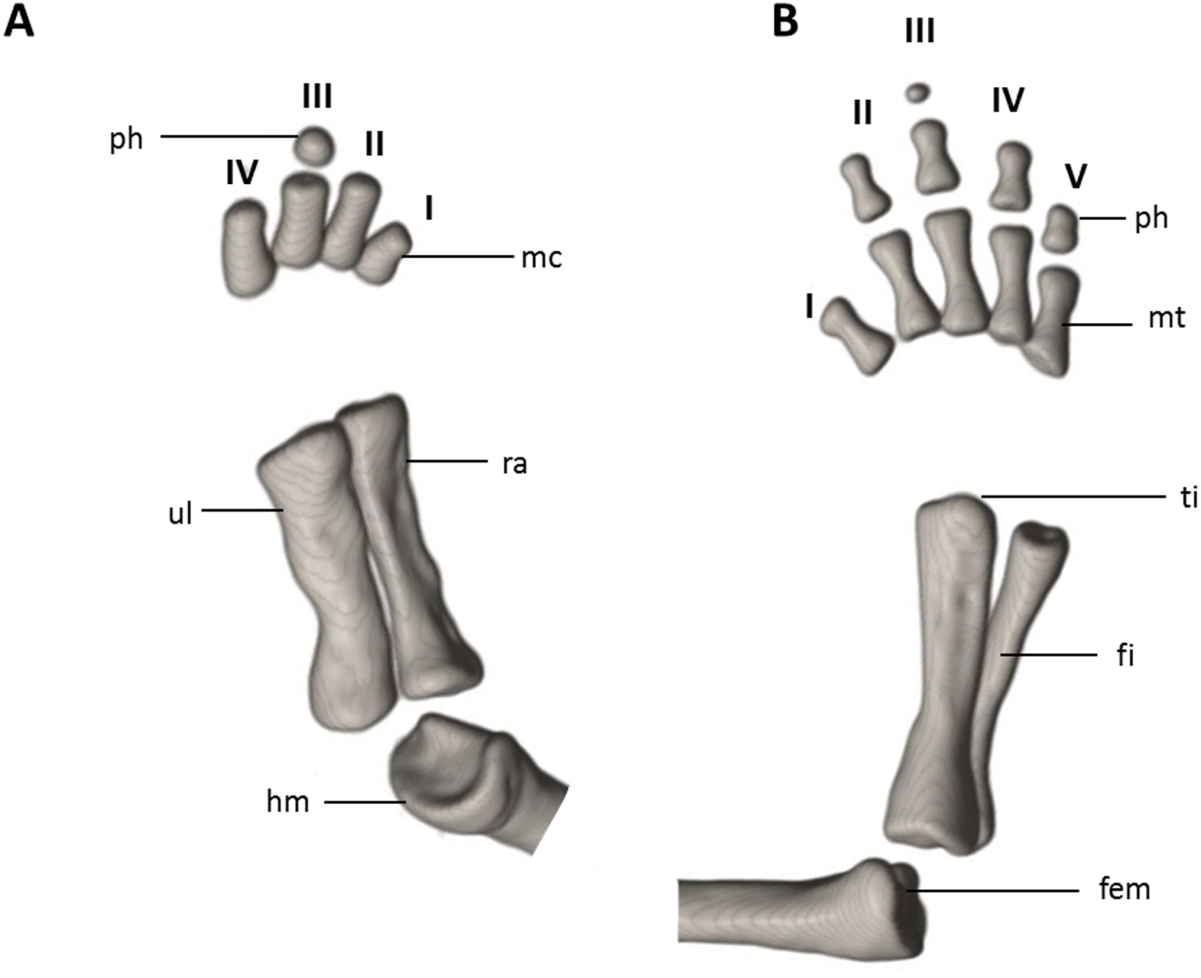
Osteology of the limbs of *Oedipina ecuatoriana* sp. n. (holotype, BMNH 1901.3.29.115, SL =45.6 mm). The (A) left forelimb and (B) the right foot is shown in dorsal aspects. Digits numbered I–V. fem, femur; fi, fibula; hm, humerus; mc, metacarpalia; mt, metatarsalia; ph, phalanges; ra, radius; ti, tibia; ul, ulna.

**Distribution and natural history.** *Oedipina villamizariorum* inhabits Evergreen lowland forests of the Ecuadorian Chocó (sensu MAE 2013). The new species is known only from one specimen collected at the type locality on the western foothills (920 m) of the Andes in the province of Carchi, Ecuador. The holotype was collected during excavations along stony edges of a stream with bushy thick canopy, near forest clearings, between 10:00 and 12:00 in the morning. The specimen was found at approximately 15 cm depth inside moist humus-rich soil covered with moderate leaf litter. One individual of caecilian was found a few meters away.

**Etymology.** Named in honor of Jorge and María Teresa Villamizar of Cumbayá, Ecuador; parents of Felipe Villamizar, who has been an important supporter of forest conservation in the area where this species was discovered. The noun is the genitive plural of the last name of the family.

*Oedipina ecuatoriana* sp. n.

Figs. 1–4, 6, 11–13

LSID urn:lsid:zoobank.org:act:5DF89BF2-74FB-441D-943C-2E2908B52CF8

**Proposed standard English name:** Ecuadorian worm salamander

**Proposed standard Spanish name:** Salamandra gusano ecuatoriana

**Holotype.**—BMNH 1901.3.29.115, male, from Paramba (Hacienda Parambas?, see Remarks section), provincia de Imbabura, Ecuador (approximate coordinates 0.816667, −78.35000, 1,067 m) (Fig. 1), obtained by purchase from W.F.H. Rosenberg (see below, Remarks).

**Diagnosis.** We assign *Oedipina ecuatoriana* to the genus *Oedipina* following the characterization of Brame (1968) based on the following combination of characters: 18 trunk vertebrae; 17 costal grooves per side; sublingual fold present; body greatly attenuated; limbs small (9 costal folds exposed when limbs appressed to sides of trunk); very long tail, more than twice standard length. In addition, we assign the new species to the subgenus *Oedopinola* (García-París & Wake, 2000) on the basis of having 18 trunk vertebrae.

*Oedipina ecuatoriana* is of moderate size for the genus (SL 45.6 mm) with a narrow head (4.9 mm HW) and a broadly rounded snout. The new species differs from all known *Oedipina* by the presence of paired prefrontal bones unique to this species in the entire genus (Wake, 1966) and a reduced phalangeal formula: 0-0-1-0; 0-1-2-1-1. *Oedipina ecuatoriana* differs from *O. parvipes* by having a broadly rounded rather than pointed snout, by having short, blunt-tipped rather than longer and pointed digits, and by having much more extensive white pigment on the head. It differs from *O. complex sensu stricto* by its larger size, by having a longer snout that is rounded rather than blunt-tipped, and shorter and more robust, rounded digits rather than weak and flattened, and often longer digits. The new species was once assigned (Dunn, 1926) to *O. parvipes,* from which it differs by having a blunter and shorter snout, more extensive white pigmentation of the head, and shorter, blunter digits. *Oedipina ecuatoriana* is distinguished from *O. maritima* in having 17 versus 0-8 maxillary teeth, and it generally differs from other members of *Oedopinola* in the complete dorsal pigmentation of its head and abundance of maxillary teeth. *Oedipina ecuatoriana* can be distinguished from *O. berlini* (characters in parentheses) by the presence of a narrow head and a broadly rounded snout (flat head, more than twice as wide as high, with relatively large protruding eyes). *Oedipina ecuatoriana* can also be distinguished from *O. fortunensis* (characters in parentheses) by the presence of a narrow head and a broadly rounded snout (narrow head and a relatively short rounded snout). *Oedipina ecuatoriana* differs from *O. villamizariorum* by having a broadly rounded snout rather than relatively pointed head with a blunt snout, by having the tip of the digits rounded instead of pointed and, by having the tips of the Fingers 2 and 3 free rather than 2, 3 and 4 as in *O. villamizariorum*; the two species also differ in extent of white pigmentation on the head, which is much greater in *O. ecuatoriana*.

**Description of holotype (Figs. 3, 4 and 6)** (measurements and counts from Brame & Wake, 1963), with a few differences based on reexamination of the holotype by DBW, (September 20, 2011). A species of moderate size and robustness; adult male (SL 45.6 mm). The head is moderately broad and cylindrical; very broadly rounded at its tip; weakly developed nasolabial protuberances; SL 9.3 times head width. Mental gland not evident at tip of jaw. SL 6.1 times head length. Small nostrils slightly laterally directed. Eyes are relatively small and dorsolaterally oriented; slightly extending beyond the lateral margins of the head. The suborbital groove does not intersect the lip line. Mouth mutilated in preservation, but apparently no premaxillary teeth. Maxillary teeth (25) moderate in number, sharp. Vomerine teeth (19) are borne in a long V-shaped row extending under the internal nares. There are 17 costal grooves between the limbs, counting one each in the axilla and the groin (18 trunk vertebrae). Limbs are moderately short and slender; limb interval 9. Left forelimb missing. Hands and feet are small and moderately broad. The relatively broad digits are syndactylous, tip of the short digits rounded. Hand entirely rounded with no distinct digits, which are not even delimited by grooves. Only the broad tips of toes 2 and 3 are free. Toes, in order of decreasing length, 3-2-4-5-1. The tail is round, cylindrical, narrow in cross-section and moderately long; SL 0.58 times tail length.

**Measurements (in millimeters) of holotype.—**Head width 4.9; snout to gular fold (head length) 7.5; head depth at posterior angle of jaw 1.0; eyelid width 0.7; eyelid length 1.5; anterior rim of orbit to snout 2.4; horizontal orbit diameter 0.8; interorbital distance 2.3; distance separating eyelids 2.6; nostril diameter 0.1; snout projection beyond mandible 1.1; snout to posterior angle of vent (standard length) 45.6; tail length (separated from body) 79.2; axilla to groin 29.3; snout-gular fold 7.5; orbit-snout 2.4; limb interval 9; shoulder width 4.3; tail width at base 3.7; tail depth at base 3.9; forelimb length (to tip of longest digit) 5.0; hind-limb length 8.2; hand width 1.0; foot width 1.7. Numbers of teeth: premaxillary 0; maxillary 25; vomerine 32.

**Coloration of the holotype in alcohol (Fig. 6).** From Brame & Wake (1963, p. 11): “Dark brown dorsally and laterally, gray-brown ventrally; head with large white patch from anterior edge of eyes to level of gular fold, white coloration on snout; dorsum on proximal portions of fore and hind limbs whitish”. About 50 years later (September 20, 2011) color notes of DBW as follows: Extensive white pigment on head from in front of eyes over eyelids posterior to gular fold, extending along lateral margin in broad horseshoe shape. Brown pigment extends to point about 2/3 distance from gular and nuchal folds. Grooves in gular area fully white **“pigmented”** (emphasis in original, as opposed to light colored because of absence of pigment). Lower eyelid depigmented. Small white patch on tip of snout; larger ones surround each nostril. Forelimb with white pigment on proximal base. Hind limb with white pigment on proximal base. Rich brown dorsally, much paler ventrally, with color broken into punctate melanophores. Small white patches of irregular shape on tail and sides of body.

**Osteology.** The following account is based on a micro CT scan of the holotype, male BMNH 1901.3.29.115 (Figs. 11–13). The skull is robust and well ossified. Anterior cranial elements are especially well-formed. The snout is relatively long and well developed and is broadly rounded in outline. The premaxilla is a single bony rod with a slightly expanded and somewhat offset pars dentalis that lacks teeth; the pars dentalis is well separated from each maxilla. The posterodorsal tip of the bone ends in a rounded tab that articulates with the facial portions of the combined frontal bones. The premaxilla is sunken between the large, paired, well-developed and protuberant nasals. These bones are as large or larger than any observed in members of the genus. The nasals are well separated along the dorsal midline. They are generally quadrangular with a posterior portion that articulates with the facial portion of the frontals. This portion of the bone is excised laterally to provide passage for the nasolacrimal duct; a less evident impression is present on the neighboring prefrontal bones, so the duct passes between the posterior margin of the nasal and the dorsal prefrontal. The paired prefrontals, unique to this species in the entire genus (Wake, 1966), are quadrangular in shape and well-formed. They articulate with the facial process of the maxilla ventrolaterally and partially with the nasal dorsoanteriorly. They are well separated from the frontals. Septomaxillae are absent and the nasals and large maxillary facial lobes occupy the vacated area. The maxillae are relatively short and stout; they terminate at approximately the center of the orbit. There are 10 teeth on the left maxilla and 15 on the right maxilla (counts based on counts of intact specimen, not scans). The facial lobe of the maxilla is well anterior to the midpoint of the toothed portion and strongly articulates with the prefrontal; there is no contact with the nasal. The frontals and maxillary facial lobes are not in contact. A small internasal fontanelle is present at the level of the anterior margin of the orbit; it is enveloped by the frontal bones, which articulate both anteriorly and posteriorly around it. Frontoparietal fontanelle absent. Frontals are broad along most of their length, tapering rapidly in front of the internasal fontanelle. The frontals are well articulated to one another medially, to frontal processes of the premaxilla anteriorly, to nasals anterolaterally, to underlying orbitosphenoids laterally, and to parietals posteriorly. The suture of the frontals with the parietals is a nearly straight line dorsally. Posteriorly frontals do not reach the posterior margins of the orbitosphenoids. The parietals are more or less rectangular and have a posterolateral depression for passage of the adductor mandibulae muscles. They are well articulated to one another medially, to orbitosphenoids laterally, to otic-occipitals posteriorly, and to frontals anteriorly. The squamosal is oblique and articulates firmly with the otic-occipital and the quadrate. There is no indication of a posteriorly directed process or spur on the squamosal, usually seen in the genus. The quadrate is massive. The operculum is a simple, round disk and lacks a stylus. The occipital condyles are short and stout. Ventrally the paired vomers are large and well developed and are separated along the midline for their entire length, forming a narrow, elongated fontanelle. They articulate anteriorly with the maxillae, posterodorsally with the orbitosphenoid, and posteriorly with the parasphenoid. The preorbital processes of the vomers are relatively stout and long, coming to a point that extends laterally, beyond the lateral margins of the body of vomers and approach maxillae. Each vomer bears a series of teeth that cannot be counted in the scans but collectively number 32 in the intact specimen. About 60 - 70 articulated teeth are present in each broad posterior vomerine patch. The patches are joined along the midline both anteriorly and posteriorly with a space in between. The orbitosphenoids are well developed and articulate dorsally with the frontals and parietals, ventrally with the parasphenoid, and anteroventrally with the vomers. The optic fenestrae are enclosed in bone and are located in the posterior one-third of the bone. The parasphenoid is long but does not reach the anterior margin of the orbitosphenoid. It is oval-shaped posteriorly and tapers slowly anteriorly, where it terminates above the vomers. Anteriorly the parasphenoid overlaps with the vomers, articulates strongly with the orbitosphenoids laterally, and with the otic-occipitals laterally and posteriorly. The lower jaw is well developed but accidentally separated at the mandibular symphysis. The dentary is long and simple in structure and bears a series of teeth that is uncountable. The prearticular is relatively large and robust and envelopes the apparently mineralized articular cartilage.

The hyobranchial apparatus is invisible and apparently formed entirely of unmineralized cartilage.

The vertebral column includes 1 atlas, 18 trunk, 1 sacral, 2 caudosacral, and a long series of caudal vertebrae in a long tail that greatly exceeds SL and is unbroken at the tip but separated from the body. The atlas is fully ossified and distinctly shorter than the trunk vertebrae. The ribs are very short. No details are presently available for the posterior trunk and tail skeleton, except for the hind limbs.

Limbs relatively robust. No tibial spur visible. The phalangeal formulae are 0-0-1-0 for the hand and 0-1-2-1-1 for the foot.

**Distribution and natural history.** *Oedipina ecuatoriana* presumably inhabits Evergreen lowland mountain forests of Ecuadorian Chocó (sensu MAE, 2013). The holotype was obtained by professional collectors at “Paramba”, which we assume is near the present-day Hacienda Paramba, in the western foothills of the province of Imbabura. This is the southernmost locality of the genus *Oedipina*. The species is known only from the holotype and no further details are available (See Remarks).

**Etymology.** The name “ecuatoriana” honors the country of Ecuador, an important center of amphibian biodiversity.

### Remarks concerning Ecuadorian specimens

The unique specimen of *Oedipina ecuatoriana* from Paramba was part of a collection sold to the British Museum (Natural History) (= The Natural History Museum, London) by the naturalist, collector and natural history dealer W. F. H. Rosenberg (The Natural History Museum archives; DF Cisneros-Heredia, pers. obs., 2013). William Frederick Henry Rosenberg (born 1868, died 1957) collected in western Ecuador from November 1896 to September 1897, but his colleagues Georg Flemming and Rudolf Miketta kept sending him collections after his departure—at least until 1903 (Jobling, 2019; Lemaire & Venedictoff, 1989; Rosenberg, 1898; Sharpe, 1906). Either Flemming or Miketta most probably collected the specimen of *O. ecuatoriana*. Little information is available about these two collectors: Georg Flemming (birth and death dates unknown) was probably related (son?) to Bernhard (Bernardo) Flemming, a German engineer who was hired at different times along the late 1800s for the construction of the road between Ibarra and El Pailón (San Lorenzo del Pailón), passing through Paramba (Flores, 1892; Rueda Novoa, 2010), and who in 1891 published the map “Mapa general del Ecuador por Bernardo Flemming”, where Paramba is shown (Flemming, 1891). Georg Flemming lived in (or visited) Paramba at least until 1907, based on a letter sent to the German journal for bookbinders “Archiv für Buchbinderei” dated “Paramba, 6 Februar 1907” (Flemming, 1907). Rudolf Miketta (born 1867; died unknown) lived in Ecuador and apparently lived and died in the town of Bahía de Caraquez (Ceriola, 1913).

Data on the historic collecting locality of Paramba is incomplete, thus we provide additional information herein. Boulenger (1898) and Hartert (1898) cited a description of Paramba provided by Rosenberg (the latter being more detailed and presented herein): “a farm on the western bank of the River Mira. Its elevation is 3,500 ft altitude [ = 1,067 m], and it is still in the forest region, but the open country commences two or three miles higher up the Mira. Sugar, rice, maize, cacao, and coffee are all cultivated on the various farms in this district. The city of Ibarra [is], two days’ ride from Paramba, and about the same distance from Quito”. Although the locality “Paramba” has usually been regarded as a single “hacienda” (landed estate) (Lynch & Duellman, 1997), the description by Rosenberg points to the existence of “various farms in this district”. We have found references to apparently two different haciendas called Paramba, one owned by the Zaldumbide family since 1857 (Jácome Clavijo, 2007; Morales Almeida, 1960), and a second one owned by a “Mr. Flemming” around 1919—possibly Bernhard Flemming (Franco, 1919).

The haciendas of Paramba were located on the mountains of Malbucho, western side of the River Mira, province of Imbabura (Jácome Clavijo, 2007), and probably took their name from the nearby River Paramba (Villavicencio, 1858). Access to the region was possible through the “Camino de Malbucho” or “Camino de Carondelet”, which connected Quito and Ibarra with El Pailón on the Pacific lowlands, but that reached only as far as Paramba during the XIX century—where Bernhard Flemming worked on several occasions (Anonymous, 1895). After Rosenberg, Flemming and Miketta, several other naturalists and collectors explored Paramba. During the 20th century, the road became part of the Ibarra-San Lorenzo railroad (Fierro, 1894) and the town of Parambas was established, probably on lands of one of the haciendas.

Bristow (1979) suggested Rosenberg’s data were not precise and Paramba was a region centre. Bristow saw Rosenberg’s report that he collected during the dry season as erroneous, considering that Rosenberg’s travels took place during the rainy season. However, Rosenberg and his associates were in Paramba across the year, not just the dry season, and any weather inconsistency could be the result of impacts by El Niño Southern Oscillation event of 1886–1887 (Wolter & Timlin, 2011). Bristow also remarked that all specimens dealt from Paramba have the same elevation reported (3,500 ft), which is unlikely, and probably corresponds to the elevation of the hacienda house, taken as a fixed reference point, while collections were obtained from nearby on the surrounding hills and in ravines. Ayala & Williams (1988) suggested that Paramba specimens could have been collected as far as 20 km away from the hacienda house, but it seems unlikely since nearby localities were distinguished and properly named (e.g., Cachiyacu). The exact location of the Hacienda Paramba of Rosenberg is difficult to establish with precision.

We examined a specimen of *Oedipina* (FHGO 9642) from Padre Santo, Playa de Oro (0.867353, −78.804113, 130 m), Esmeraldas, Ecuador. The specimen was collected by Manuel Morales-Mite in 2000 at 130 m of elevation and deposited at the Fundación Herpetológica Gustavo Orcés. The specimen FHGO is similar to the holotype of *Oedipina villamizariorum*. Both FHGO 9642 and DHMECN 14489 differ more from *Oedipina ecuatoriana* sp. n. (BM 1901.3.29.115) than from each other (Figs. 4, 6 and 10). We think the FHGO specimen represents a species closely related to *O. villamizariorum*, but we lack conclusive evidence. The phalangeal formulae of the hands of two osteological preparations differ. While phalanges appear to be erratically lost in *Oedipina*, and likely display some intraspecific variation, we are reluctant to make too much of the difference noted (FHGO has one fewer phalanx in one digit). Digits in general are frequently reduced in complexity in this genus and the differences are unlikely to represent positively selected traits or directly adaptive traits. We know that the two specimens are adults, as shown by the fully articulated frontals and parietals and absence of a fontanelle between these bones (Fig. 10). However, the two specimens also differ with respect to whether the atlantal pedicels are fused (DHMECN 14489) or remain separated (FHGO 9642) over the nerve cord. We think this just could be a relative degree of maturity and with no diagnostic value.

The snout is more rounded, less “pinched” in FHGO 9642. This characteristic could well turn out to be important, but the low sample number of *Oedipina* from Ecuador prevents us from making more detailed assertions. There are some small differences in the shapes of the squamosals; the squamosal spur is a bit more evident in FHGO 9642, but it is still far less prominent than in Central American species.

Geographically *Oedipina villamizariorum* is known from 925 m of elevation in the province of Carchi and *O. ecuatoriana* from 1,067 m in the province of Imbabura and they represent different species even though the distance between them is only 23.6 km in straight line. Specimen FHGO 9642 is known from the lowlands at 130 m of elevation in Padre Santo Esmeraldas province, and distances between its locality and other Ecuadorian *Oedipina* are 50 to 63 km in straight line (Fig. 1). In that sense, we are aware that this is a substantial range for a tropical salamander but not unprecedented for *Oedipina* (Wake, 1987). We strongly suspect FHGO 9642 is a third species for Ecuador, but we refrain from describing it at this time. We include comparative information, preserved images and CT-scan images for future researchers (Figs. 4, 6 and 10). The specimen (FHGO 9642) was found on the top of a large leaf in herbaceous vegetation, perched approximately 1.5m high above the ground. The specimen was still but attentive when the collector approached to capture it. It jumped and “dived” through the vegetation to the ground (Morales-Mite, pers. comm., 2004).

### Extinction risk

*Oedipina villamizariorum* and *O. ecuatoriana* each are known from two provinces, with just three records from Ecuador for the genus. We inferred, based on geographical data, deforestation statistics (Sierra, 2013; SUIA, 2020) and distribution of current and projected mining concessions (ARCOM, 2020; INIGEMM, 2020), that continuous decline of its habitat quality in Carchi and Imbabura has occurred over the last 10 years and is expected to continue in the future. We suggest that *O. villamizariorum* and *O. ecuatoriana* should be classified under the IUCN category Critically Endangered (CR B1ab (i, iii)).

## Discussion

All known records of *Oedipina* within Ecuador (i.e., *O. villamizariorum* and *O. ecuatoriana* and *O.* aff. *villamizariorum*) come from a small area in the northwest, between the provinces of Carchi, Esmeraldas and Imbabura. Although the two new species may be able to live under anthropogenic impacts (at least to some degree), they probably depend on remaining forest patches to survive, especially given that fossorial species are affected by soil changes following forest loss (Brodman et al., 2006). *Oedipina villamizariorum* and *O. ecuatoriana* are likely rare species, with only one specimen for each species ever collected. Habitat change, fragmentation, and loss in the region is extensive and continues due to the expansion of the agricultural frontier and mining (Guayasamin et al., 2019; Roy et al., 2018; Sierra, 2013). Intensive herpetological surveys across the provinces of Esmeraldas, Imbabura, and Carchi have been conducted (MECN, 2013; Yánez-Muñoz et al., 2010; Yánez-Muñoz et al., 2018; Yánez-Muñoz et al., 2020) however, no *Oedipina* have been recorded. Given the fossorial habits of the animals, specific survey methods will likely be required to encounter them with any frequency; they may only appear to be rare. We have included all sampling effort carried out in the type localities and nearby areas (i.e., 2,300 hours/80 persons). In these current and historical surveys, standardized methods for searching amphibians have been applied, such as litter removal and under-log search. However, digging has been less used and therefore the sampling effort presented cannot be exclusively attributed to the specific search for *Oedipina*. It is important to mention that one of the documented specimens in Ecuador was observed 1.5 m above the ground in herbaceous vegetation. Thus, natural history data of the genus is fragmentary.

*Oedipina ecuatoriana* was assigned to *O. parvipes* by Dunn (1926), in an addendum to his monograph. He noted the “large white patch on the top of the head from between the eyes to the neck”, unusual for the species, and also noted that the specimen is darker than usual for *O. parvipes.* Because he described *O. complex* (Dunn, 1924), his decision is important because he considered BMNH 1901.3.29.115 to be distinct from *O. complex*. *Oedipina ecuatoriana* has a relatively long and rounded snout, not short and blunt as in *O. complex*. Additionally, *O. ecuatoriana* is larger than any available specimens of *O. complex*. Furthermore, none of Dunn’s material of *O. complex* is reported to have a white or even lightly colored head. Nevertheless, the specimen was subsequently assigned to *O. complex* by Brame & Wake (1963). The latter authors provided detailed measurements of the specimen and noted that it was a “very large” specimen (in fact, the largest of any specimen yet assigned to *O. complex*). We note that the holotype of *O. ecuatoriana* is a male, the smaller of the typically sexually dimorphic tropical plethodontids (García-París & Wake, 2000; Wake, 1993). Salient features leading to this assignment of Brame & Wake (1963) were the rounded snout, considered intermediate between the pointed snout of *O. parvipes,* as then understood, and the blunt snout of typical *O. complex*, the large number of maxillary teeth (they reported 17, DBW later counted 25), typical of topotypic *O. complex* but found only in the holotype (they report 19) of *O. parvipes* (a topotypic adult has only 2 maxillary teeth; Brame, 1968). However, they found more anterior vomerine teeth than in specimens assigned to either species (32, in patches in *O. complex*). The foot of *O. ecuatoriana* was reported to be less syndactylous and to have shorter and broader digits than *O. complex*, and it also differs from *O. parvipes* in this respect. Additionally, Brame & Wake (1963) stated that both *O. complex* and *O. parvipes* are known to have white heads. In short, the assignment was tenuous.

A table of measurements of ten specimens each of both *O. parvipes* and *O. complex* was presented by Brame (1968). This included six specimens of topotypic *O. complex* from Barro Colorado Island, Panama, and these ranged in SL from 38.2–40.5 mm. A near topotypic female from Fort Sherman, Panama, was found to be 43.1 mm SL and is the largest specimen, if it is properly assigned to *O. complex.* The eyes of *O. ecuatoriana* are much more prominent than topotypic *O. complex*. *Oedipina ecuatoriana* also has more prominent digits than *O. complex*. Observations (by DBW) on additional species from Panama and Colombia suggest that most species of *Oedipina* are geographically restricted, like those in Central American countries, and a modern revision, which uses molecular as well as morphological data, is badly needed. At present fresh tissue samples for such a study are lacking. An example of the complexity is that Cerro Campana, just a short distance north and west of the Panama Canal, is home to a population that is larger, darker in coloration, and molecularly distinct from topotypic *O. complex* (Fig. 2). Panamanian populations from the province of Cocle (e.g., Valle de Antón and El Cope), further west, may represent yet another species, and the population reported by Dunn (1926) from La Loma in the province of Chiriqui, far to the west, and assigned to *O. parvipes* likely represents another unnamed species. Similar situations prevail in northwestern Colombia.

If we restrict comparisons of the Ecuadorian specimens to topotypic *O. complex*, we note that the Ecuadorian specimens (both *O. villamizariorum* and *O. ecuatoriana*) are larger and more robust, with a distinctly longer snout, and they have a prominent, pigmented patch of white on the snout and over much to all of the head and neck. Our limited genetic data suggest a close relationship of *O. villamizariorum* to *O. complex*, a surprising result given the great geographic separation. While we think the species in Ecuador are distinct, they may well be relatively recent arrivals in the region.

Our initial thought was that the specimen of *O. villamizariorum* from Carchi was a second specimen of the species known from Imbabura (=BMNH 1901.3.29.115). Differences in snout shape and structure of the hands and feet first suggested the possibility that two species were involved, and the skeletal scan provided conclusive evidence. The presence of paired prefrontal bones in *O. ecuatoriana* is unique in the genus (and not present in *O. villizariorum* and in any other species of *Oedipina*), and there are numerous other skeletal differences, including the structure of the hands and feet and the strongly reduced phalangeal formulae of *O. ecuatoriana* that differentiate it from *O. villizariorum*. The basic phalangeal formula for *Oedipina* is 1-2-3-2 and 1-2-3-3-2 (Brame, 1968; García-París & Wake, 2000; Wake, 1966) but there are numerous divergences among species. Within *Oedopinola* several instances of substantial reduction are recorded. The recorded formula for *O. complex* is 0-1-2-1 and 0-1-2-2-1 ; the specimen from Fort Sherman questionably assigned to *O. complex* has a formula of 1-2-2-1 and 0-2-2-2-1 . Our new species *O. villamizariorum* is somewhat intermediate with 0-1-2-1 and 0-2-2-2-1 . The holotype of *O. savagei* has a formula of 0-1-1-1 and 0-1-2-1-1 , so is more reduced than any of the above, and *O. nimaso* (Boza-Oviedo et al., 2012) has also more reduced: 0-1-2-1; 0-1-2-1-1. The holotype of *O. fortunensis* is 0-1-2-1 and 0-1-2-2-1 (Köhler, Ponce & Batista, 2007). The formula for *O. maritima* (modal) is 0-1-2-1; 1-2-2-2-1. However, no species of *Oedipina* shows a reduction as great as *O. ecuatoriana*, which has the greatest reduction of any plethodontid salamander: 0-0-1-0; 0-1-2-1-1.

The character of paired prefrontal bones in *Oedipina ecuatoriana* is an extremely important morphological character and has not been reported in any member of the genus (García-París & Wake, 2000; Wake, 1966). *Oedipina* seems to be rare and difficult to collect, as reflected in the few specimens registered from Ecuador. Based on our experience we recommend that specimens collected in the future be studied with CT-Scan image analysis in addition to DNA samples. Specimens of *O. ecuatoriana* would be easily identifiable by the presence of prefrontal bones, a unique character in the entire genus. We assign this species (*O. ecuatoriana*) within the genus based on morphological synapomorphies. On the other hand, while the specimen from Padre Santo, Esmeraldas, presents an external morphology similar to *O. villamizariorum*, there are differences in the shape of hands, feet and osteology that restrict us from assigning the specimen to any known species. We consider it to be a potential new species related to *O. villamizariorum*.

The fossorial nature of *Oedipina*, and the biogeographic history and evident low dispersal capacity of these small vertebrates can in the future help explain the existing diversity of the group. Currently, the lack of genetic material of topotypical species is the main issue that should be addressed. Based on this information we emphasize targeted searches for *Oedipina* within Ecuador, mainly in type localities and areas nearby type localities of the species described herein.

## Conclusions

We provide morphological, osteological and genetic evidence (for *Oedipina villamizariorum*) that validates the description of two new species of extremely rare salamanders, *O. villamizariorum* sp. n. and *O. ecuatoriana* sp. n. We found three specimens of *Oedipina* within Ecuadorian territory in museum collections, two of which we describe as new species, but we refrain from assigning the third, since further study is pending.

*Oedipina ecuatoriana* is the first species of *Oedipina* with the presence of **paired prefrontal bones** and a greatly reduced phalangeal formula: 0-0-1-0; 0-1-2-1-1. Finally, we present a phylogenetic hypothesis for the genus, including one of the two new species.

##  Supplemental Information

10.7717/peerj.9934/supp-1 Data S1 Examined specimens of *Oedipina*

10.7717/peerj.9934/supp-2 Supplemental Information 2 DNA sequences and accession codes used

10.7717/peerj.9934/supp-3 Supplemental Information 3 Aligned DNA sequence matrix for phylogenetic analyses

## Additional Information and Declarations

### Field Study Permissions

The following information was supplied relating to field study approvals (i.e., approving body and any reference numbers): The fieldwork and the collection of specimens was approved by Ministerio del Ambiente (MAE), Ecuador (MAE-DNB-CM-2016-0045 and Nº018-2017-IC-FAU-DNB/MAE). We conducted this research under permits MAE–DNB–CM–2018–0106 and MAE-DNB-CM-2019-0120, issued by the Ministerio del Ambiente del Ecuador.

### DNA Deposition

The following information was supplied regarding the deposition of DNA sequences: The nucleotide sequences are available in a Supplemental File and at GenBank: MT328210 (*Oedipina villamizariorum* DHMECN14489); MT329630 (Oedipina villamizariorum BankIt2332790 DHMECN14489_QCAZ75501). The aligned matrix is available at Zenodo: Santiago Ron. (2019). Aligned DNA sequence matrix for phylogenetic analyses in the article “New species of fossorial salamanders of the genus *Oedipina* (Plethodontidae) from the northwestern Ecuador” (Version 1.1) [Data set]. Zenodo. http://doi.org/10.5281/zenodo.3628754.

### Data Availability

The following information was supplied regarding data availability: The DNA sequences, accession numbers, and an appendix of examined specimens in museum collections are available in the Supplemental Files.

### New Species Registration

The following information was supplied regarding the registration of a newly described species: Publication LSID: urn:lsid:zoobank.org:pub:1B4C2057-0FFC-4DAE-9267-240AD991EC7C *Oedipina villamizariorum* LSID: urn:lsid:zoobank.org:act:A47B831D-EF23-44FC-929D-E7575A50F1DF *Oedipina ecuatoriana* LSID: urn:lsid:zoobank.org:act:5DF89BF2-74FB-441D-943C-2E2908B52CF8

## References

[ref-1] Acosta-Galvis AR, García-Cobos D, Cárdenas-Arévalo G, Corrales-Garcia A, Paternina-Hernández A (2020). Geographic distribution extension of the Worm Salamander, *Oedipina complex* (Dunn, 1924), in the Magdalena Valley, Colombia. Check List.

[ref-2] AmphibiaWeb (2020). https://amphibiaweb.org.

[ref-3] (1895). El Camino al Pailon y los ingleses. El Pichincha, diario radical de la mañana Quito. 2.

[ref-4] ARCOM (2020). Estadística Minera Actualizada. http://www.controlminero.gob.ec/.

[ref-5] Ayala SC, Williams EE (1988). New or problematic *Anolis* from Colombia. VI. Two fuscoauratoid anoles from the Pacific lowlands, *A. maculiventris* (Boulenger, 1898) and *A. medemi*, a new species from Gorgona island. Breviora.

[ref-6] Boulenger GA (1898). An account of the reptiles and batrachians collected by Mr. W. F. H. Rosenberg in western Ecuador. Prodeedings of the Zoological Society of London.

[ref-7] Boza-Oviedo E, Rovito SM, Chaves G, Garcća-Rodríguez A, Artavia LG, Bolaños F, Wake DB (2012). Salamanders from the eastern Cordillera de Talamanca, Costa Rica, with descriptions of five new species (Plethodontidae: Bolitoglossa, Nototriton, and Oedipina) and natural history notes from recent expeditions. Zootaxa.

[ref-8] Brame AH (1968). Systematics and evolution of the Mesoamerican salamander genus *Oedipina*. Journal of Herpetology.

[ref-9] Brame AH, Wake D (1963). The salamders of South America. Countributions in Science, Los Angeles County Museum.

[ref-10] Bristow CR (1979). *Opsiphanes quiteria angostura* a new sub-species of Brassolinae (Lepidoptera; Rhopalocera) from western Ecuador, with notes on collectors and localities. Journal of Natural History.

[ref-11] Brodie ED, Acevedo M, Campbell JA (2012). New salamanders of the genus *Oedipina* (Caudata: Plethodontidae) from Guatemala. Journal of Herpetology.

[ref-12] Brodman R, Parrish M, Kraus H, Cortwright S (2006). Amphibian biodiversity recovery in a large-scale ecosystem restoration. Herpetological Conservation and Biology.

[ref-13] Ceriola JB (1913). Manabói a la vista.

[ref-14] Chernomor O, Von Haeseler A, Minh BQ (2016). Terrace aware data structure for phylogenomic inference from supermatrices. Systematic Biology.

[ref-15] Crawford AJ, Lips KR, Bermingham E (2010). Epidemic disease decimates amphibian abundance, species diversity, and evolutionary history in the highlands of central Panama. Proceedings of the National Academy of Sciences of the United States of America.

[ref-16] Darda DM, Wake DB (2015). Osteological variation among extreme morphological forms in the Mexican salamander genus *Chiropterotriton* (Amphibia: Plethodontidae): morphological evolution and homoplasy. PLOS ONE.

[ref-17] Deban SM, Scales JA, Bloom SV, Easterling CM, O’Donnell MK, Olberding JP (2020). Evolution of a high-performance and functionally robust musculoskeletal system in salamanders. Proceedings of the National Academy of Sciences of the United States of America.

[ref-18] Duméril AMC, Bibron G, Duméril AH (1854). Erpétologie Genérale ou Histoire Naturelle Complète des Reptiles.

[ref-19] Dunn ER (1924). New salamanders of the genus *Oedipus*: with a synoptical key. Chicago: Field Museum of Natural History.

[ref-20] Dunn ER (1926). The salamanders of the family Plethodontidae. Smith College Fiftieth Annals Publications Northampton, Massachusetts.

[ref-21] Fierro V (1894). Informe del Sr. Gobernador de Imbabura al Ministro de Obras Públicas. Informe del Ministro de Obras y Crédito Públicos al Congreso Constitucional de 1894, Quito.

[ref-22] Flemming B (1891). Mapa general del Ecuador por Bernardo Flemming.

[ref-23] Flemming G (1907). Deutsche Bücher an der Westküste. Archiv für Buchbinderei.

[ref-24] Flores A (1892). Mensaje del Presidente de la República del Ecuador, Congreso Ordinario de 1892. Quito.

[ref-25] Franco E, Oficios TDlEdAy (1919). Ferrocarril de esmeraldas a Quito: rectificaciones a las rectificaciones.

[ref-26] Frost DR (2020). http://research.amnh.org/vz/herpetology/amphibia/.

[ref-27] García-París M, Wake DB (2000). Molecular phylogenetic analysis of relationships of the tropical salamander genera *Oedipina* and *Nototriton*, with descriptions of a new genus and three new species. Copeia.

[ref-28] Guayasamin JM, Cisneros-Heredia DF, Vieira J, Kohn S, Gavilanes G, Lynch RL, Hamilton PS, Maynard RJ (2019). A new glassfrog (Centrolenidae) from the Choco-Andean Rio Manduriacu Reserve, Ecuador, endangered by mining. PeerJ.

[ref-29] Guindon S, Gascuel O (2003). A simple, fast and accurate method to estimate large phylogenies by maximum-likelihood. Systematic Biology.

[ref-30] Hanken J (1984). Miniaturization and its effects on cranial morphology in plethodontid salamanders, genus *Thorius* (Amphibia: Plethodontidae). I. Osteological variation. Biological Journal of Linnean Society.

[ref-31] Hanken J, Wake DB, Savage JM (2005). A Solution to the large black salamander problem (genus *Bolitoglossa*) in Costa Rica and Panamá. Copeia.

[ref-32] Hartert E (1898). On a collection of birds from north-western Ecuador, collected by Mr. W. F. H. Rosenberg. Novitates Zoologicae.

[ref-33] Heyer R, Donnelly MA, Foster M, McDiarmid R (2014). Measuring and monitoring biological diversity: standard methods for amphibians.

[ref-34] Hilton WA (1946). Salamanders from Barro Colorado Island, Canal Zone. Journal of Entomology and Zoology.

[ref-35] INIGEMM (2020). Biblioteca. http://www.geoenergia.gob.ec/.

[ref-36] Jácome Clavijo J (2007). Tras las huellas de Montalvo (Edición póstuma).

[ref-37] Jaramillo AF, De La Riva I, Guayasamin JM, Chaparro JC, Gagliardi-Urrutia G, Gutiérrez RC, Brcko I, Vilà C, Castroviejo-Fisher S (2020). Vastly underestimated species richness of Amazonian salamanders (Plethodontidae: *Bolitoglossa*) and implications about plethodontid diversification. Molecular Phylogenetics and Evolution.

[ref-38] Jobling JA, Del Hoyo J, Elliott A, Sargatal J, Christie DA, De Juana E (2019). Key to scientific names in Ornithology. Handbook of the birds of the world alive.

[ref-39] Kalyaanamoorthy S, Minh BQ, Wong TKF, Von Haeseler A, Jermiin LS (2017). ModelFinder: fast model selection for accurate phylogenetic estimates. Nature Methods.

[ref-40] Katoh K, Standley DM (2013). MAFFT Multiple Sequence Alignment Software Version 7: improvements in performance and usability. Molecular Biology and Evolution.

[ref-41] Keferstein WM (1868). Ueber einige Batrachier aus Costarica. Archiv für Naturgeschichte Berlin.

[ref-42] Kessing B, Croom H, Martin A, McIntosh C, McMillan WO, Palumbi SP (1989). The simple fool‘s guide to PCR. Version 1.0 ed. University of Hawaii: Special publication. Honolulu, Department of Zoology.

[ref-43] Köhler G (2011). Amphibians of Central America.

[ref-44] Köhler G, Ponce M, Batista A (2007). A new species of worm salamander (genus *Oedipina*) from Fortuna, western central Panama (Amphibia, Caudata, Plethodontidae). Senckenbergiana Biologica.

[ref-45] Kubicki B (2016). A new species of salamander (Caudata: Plethodontidae: *Oedipina*) from the central Caribbean foothills. Mesoamerican Herpetology.

[ref-46] Lemaire C, Venedictoff N (1989). Catalogue and biogeography of the Lepidoptera of Ecuador I—Saturniidae with a description of a new species of *Meroleuca* Packard. Bulletin of the Allyn Museum.

[ref-47] Lynch JD, Duellman WE (1997). Frogs of the genus Eleutherodactylus (Leptodactylidae) in western Ecuador: systematic, ecology, and biogeography.

[ref-48] Maddison WP, Maddison DR (2014). http://mesquiteproject.org.

[ref-49] McCranie J, Vieites DR, Wake D (2008). Description of a new divergent lineage and three new species of Honduran salamanders of the genus *Oedipina* (Caudata, Plethodontidae). Zootaxa.

[ref-50] MECN (2013). Herpetofauna en áreas prioritarias para la conservación: El sistema de reservas Jocotoco y Ecominga.

[ref-51] Morales Almeida R (1960). Julio Zaldumbide Gangotena (1833-1881). Poetas románticos y neoclásicos.

[ref-52] Morales-Mite MA (2004). Dinámica poblacional de las comunidades de anfibios y reptiles de siete localidades de la zona de amortiguamiento de la reserva ecológica Cotacachi-Cayapas, Esmeraldas, Ecuador BSc. Universidad del Azuay.

[ref-53] Moritz C, Schneider CJ, Wake DB (1992). Evolutionary relationships within the *Ensatina eschscholtzii* Complex confirm the ring species interpretation. Systematic Biology.

[ref-54] Mueller RL, Macey JR, Jaekel M, Wake DB, Boore JL (2004). Morphological homoplasy, life history evolution, and historical biogeography of plethodontid salamanders inferred from complete mitochondrial genomes. Proceedings of the National Academy of Sciences of the United States of America.

[ref-55] Nguyen LT, Schmidt HA, Haeseler Avon, Minh BQ (2015). IQ-TREE: a fast and effective stochastic algorithm for estimating maximum-likelihood phylogenies. Molecular Biology and Evolution.

[ref-56] Parra Olea G, Garcia-Castillo MG, Rovito SM, Maisano JA, Hanken J, Wake DB (2020). Descriptions of five new species of the salamander genus *Chiropterotriton* (Caudata: Plethodontidae) from eastern Mexico and the status of three currently recognized taxa. PeerJ.

[ref-57] Peters WCH (1879). Über neue Amphibien des Kgl. zoologischen Museums (*Euprepes*, Acontias, Typhlops, Zamenis, Spilotes, Oedipus). Monatsberichte der Königlichen Preussische Akademie des Wissenschaften zu Berlin.

[ref-58] Reyes-Puig C, Bittencourt-Silva GB, Torres-Sánchez M, Wilkinson M, Streicher JW, Maddock ST, Kotharambath R, Müller H, Angiolani Larrea FN, Almeida-Reinoso D, Cisneros-Heredia DF, Ron SR (2019a). Rediscovery of the Endangered Carchi Andean toad, *Rhaebo colomai* (Hoogmoed, 1985), in Ecuador, with comments on its conservation status and extinction risk. Check List.

[ref-59] Reyes-Puig JP, Reyes-Puig C, Ron S, Ortega JA, Guayasamin JM, Goodrum M, Recalde F, Vieira JJ, Koch C, Yanez-Munoz MH (2019b). A new species of terrestrial frog of the genus *Noblella* Barbour, 1930 (Amphibia: Strabomantidae) from the Llanganates-Sangay Ecological Corridor, Tungurahua, Ecuador. PeerJ.

[ref-60] Ron SR, Merino-Viteri A, Ortiz DA (2020).

[ref-61] Rosenberg WF (1898). Some new species of Coleoptera in the Tring Museum. Novitates Zoologicae.

[ref-62] Rovito SM, Parra-Olea G, Recuero E, Wake DB (2015). Diversification and biogeographical history of Neotropical plethodontid salamanders. Zoological Journal of the Linnean Society.

[ref-63] Roy BA, Zorrilla M, Endara L, Thomas DC, Vandegrift R, Rubenstein JM, Policha T, Ríos-Touma B, Read M (2018). New mining concessions could severely decrease biodiversity and ecosystem services in Ecuador. Tropical Conservation Science.

[ref-64] Rueda Novoa R (2010). De esclavizados a comuneros en la cuenca aurífera del Río Santiago-Río Cayapas (Esmeraldas). Etnicidad negra en construcción en Ecuador siglos XVIII-XIXPhD. Universidad Andina Simón Bolívar, Universidad Pablo de Olavide.

[ref-65] Sabaj MH (2016). Standard symbolic codes for institutional resource collections in herpetology and ichthyology: an online reference. Version 6.5 (16 August 2016). http://www.asih.org/.

[ref-66] Sambrook J, Fritsch EF, Maniatis T (1989). Molecular cloning: a laboratory manual.

[ref-67] Sanderson IT (1941). Living treasure.

[ref-68] Sharpe RB (1906). Birds. The history of the collections contained in the Natural History Departments of the British Museum.

[ref-69] Sierra R (2013). Patrones y factores de deforestación en el Ecuador continental 1990-2010. Y unacercamiento a los próximos 10 años.

[ref-70] SUIA (2020). Repositorio del Concocimiento Ambiental. uia.ambiente.gob.ec.

[ref-71] Symbiota (2020). Symbiota Promoting Bio-Collaboration. Available at http://www.symbiota.org — https://bdj.pensoft.net/articles.php?id=1114 — https://github.com/Symbiota/. http://www.symbiota.org.

[ref-72] Villavicencio M (1858). Geografía de la República del Ecuador.

[ref-73] Wake D (1966). Comparative osteology and evolution of the lungless salamanders, Family Plethodontidae. Memories of the Southern California Academy of Sciences.

[ref-74] Wake D (1987). Adaptive radiation of salamanders in Middle American cloud forests. Annals of the Missouri Botanical Garden.

[ref-75] Wake DB (1993). Phylogenetic and taxonomic issues relating to salamanders of the Family Plethodontidae. Herpetologica.

[ref-76] Wake D, Rovito SM, Maisano JA, Hanken J (2012). Taxonomic status of the enigmatic salamander *Cryptotriton adelos* (Amphibia: Plethodontidae) from northern Oaxaca, Mexico, with observations on its skull and postcranial skeleton. Zootaxa.

[ref-77] Wolter K, Timlin MS (2011). El Niño/Southern Oscillation behaviour since 1871 as diagnosed in an extended miltivariate ENSO index (MEI. ext.). International Journal of Climatology.

[ref-78] Yánez-Muñoz MH, Batallas D, Franco-Mena D, Meza-Ramos PA, Oyagata LA, Padilla D, Paucar C, Reyes-Puig JP, Rodríguez A, Urgilés-Merchán MA, Vega-Yánez M (2020). Anfibios en los Ecosistemas Andino-Tropicales de la provincia del Carchi.

[ref-79] Yánez-Muñoz M, Meza-Ramos P, Cisneros-Heredia D, Ortega-Andrade HM (2010). Serie Herpetofauna del Ecuador: El Chocó Esmeraldeño.

[ref-80] Yánez-Muñoz MH, Reyes-Puig C, Reyes-Puig JP, Velasco JA, Ayala-Varela F, Torres-Carvajal O (2018). A new cryptic species of *Anolis* lizard from northwestern South America (Iguanidae, Dactyloinae). Zookeys.

