# Supplementary material for: Two extremely rare new species of fossorial salamanders of the genus Oedipina (Plethodontidae) from northwestern Ecuador
Source: PeerJ. 2020 Oct 2;8:e9934. doi: 10.7717/peerj.9934 (PMC7534686; doi:10.7717/peerj.9934)
Supplement: Data S1 [file peerj-08-9934-s001.docx]

**Supplementary data I. Examined specimens of *Oedipina***

YPM HER A020155. *Oedipina complex.* Cocobolo Nature Reserve 250 m, Chepo, Panama Prov., Panama

LACM 134873. *O. complex*. Isla Barro Colorado, Prov. Colon, Panamá.

MVZ 236255 *O. complex.* Peninsula Bohio, Monumento Natural Barro Colorado, Prov. Colon, Panama.

KU 116672 *O. complex.* Summit Experimental Gardens, 160 m, Prov. Panama, Panama.

KU 76181. *O.* cf. *complex*. S Slope Cerro Campana, 800 m, Prov. Panama, Panama.

KU 76183. *O.* cf *complex.* El Valle del Anton, 560 m, Prov. Cocle, Panama.

BM 1929.6.1.39. *O.* cf *complex.* Ft. Sherman, Prov. Colon, Panama.
