# Supplementary material for: Two extremely rare new species of fossorial salamanders of the genus Oedipina (Plethodontidae) from northwestern Ecuador
Source: PeerJ. 2020 Oct 2;8:e9934. doi: 10.7717/peerj.9934 (PMC7534686; doi:10.7717/peerj.9934)
Supplement: Supplemental Information 2 [file peerj-08-9934-s002.docx]

Appendix 1. DNA sequences and accession codes used in this study.

| **Species** | **Voucher** | **GenBank 16S** | **GenBank cyt *b*** |
| --- | --- | --- | --- |
| *Bradytriton silus* | MVZ 265365 | KP886877 | –– |
| *Bradytriton silus* | MVZ 265366 | KP886878 | –– |
| *Oedipina alleni* | MVZ 225903 | AF199208 | AF199151 |
| *Oedipina alleni* | MVZ 190856 | –– | AF199150 |
| *Oedipina alleni* | MVZ 190857 | AF199207 | AF199149 |
| *Oedipina berlini* | UCR 22845 | KX792143 | KX792144 |
| *Oedipina capitalina* | JHT 3600 | KU495731 | –– |
| *Oedipina carablanca* | –– | FJ196862 | FJ196869 |
| *Oedipina complex* | MVZ 236255 | AF199213 | AF199157 |
| *Oedipina complex* | MVZ 233166 | AF199212 | AF199156 |
| *Oedipina cyclocauda* | MVZ 138916 | AF199214 | AF199158 |
| *Oedipina cyclocauda* | MVZ 203747 | AF199215 | AF199159 |
| *Oedipina elongata* | UTA-A 51906 | AF199216 | AF199160 |
| *Oedipina gephyra* | USNM 343462 | AF199217 | AF199161 |
| *Oedipina gephyra* | LDW 10502 | AF199218 | AF199162 |
| *Oedipina gracilis* | MVZ 210398 | AF199219 | –– |
| *Oedipina gracilis* | MVZ 203753 | –– | AF199163 |
| *Oedipina grandis* | MVZ 225904 | AF199220 | AF199164 |
| *Oedipina grandis* | MVZ 219593 | –– | AF199165 |
| *Oedipina leptopoda* | MVZ 167772 | –– | AF199193 |
| *Oedipina maritima* | MVZ 219997 | AF199221 | AF199166 |
| *Oedipina nica* | MVZ 263774 | KP886885 | –– |
| *Oedipina pacificensis* | UCR 12063 | AF199222 | AF199169 |
| *Oedipina pacificensis* | UCR E7 | –– | AF199170 |
| *Oedipina pacificensis* | MVZ 190859 | –– | AF199167 |
| *Oedipina pacificensis* | MVZ 190858 | –– | AF199168 |
| *Oedipina parvipes* | MVZ 210404 | AF199210 | AF199154 |
| *Oedipina parvipes* | MVZ 210405 | AF199211 | AF199155 |
| *Oedipina parvipes* | AJC 1786 | FJ784316 | –– |
| *Oedipina poelzi* | MVZ 194873 | AF199226 | AF199176 |
| *Oedipina poelzi* | MVZ 181235 | –– | AF199172 |
| *Oedipina poelzi* | MVZ 207128 | AF199225 | AF199175 |
| *Oedipina poelzi* | MVZ 206398 | AF199223 | AF199171 |
| *Oedipina poelzi* | –– | AY728213 | AY728213 |
| *Oedipina poelzi* | MVZ 163703 | AF199224 | AF199174 |
| *Oedipina poelzi* | MVZ 181348 | –– | AF199173 |
| *Oedipina pseudouniformis* | MVZ 181229 | –– | AF199179 |
| *Oedipina pseudouniformis* | MVZ 203749 | AF199227 | AF199178 |
| *Oedipina pseudouniformis* | MVZ 190852 | –– | AF199177 |
| *Oedipina savagei* | MVZ 229360 | –– | AF199153 |
| *Oedipina savagei* | LDG 961327 | AF199209 | AF199152 |
| *Oedipina* sp. | LDW 11270 | AF199231 | AF199192 |
| *Oedipina stenopodia* | MVZ 163649 | AF199228 | AF199181 |
| *Oedipina stenopodia* | MVZ 138918 | –– | AF199180 |
| *Oedipina taylori* | MVZ 267200 | KP886887 | –– |
| *Oedipina tomasi* | MVZ 258037 | KP886886 | –– |
| *Oedipina uniformis* | MVZ 221340 | –– | AF199182 |
| *Oedipina uniformis* | MVZ 225905 | –– | AF199183 |
| *Oedipina uniformis* | MVZ 225906 | –– | AF199188 |
| *Oedipina uniformis* | MVZ 221321 | –– | AF199189 |
| *Oedipina uniformis* | MVZ 181349 | –– | AF199191 |
| *Oedipina uniformis* | MVZ 194862 | –– | AF199186 |
| *Oedipina uniformis* | MVZ 194864 | –– | AF199187 |
| *Oedipina uniformis* | MVZ 203751 | AF199230 | AF199190 |
| *Oedipina uniformis* | MVZ 190853 | –– | AF199185 |
| *Oedipina uniformis* | MVZ 194871 | –– | AF199184 |
| *Oedipina villamizariorum* **sp. nov.** | DHMECN 14489 | MT328210 | MT329630 |
